# Synergistic Effects of Compound Dendrobium Candidum and Antihypertensive Medications on Refractory Hypertension in Spontaneously Hypertensive Rats

**DOI:** 10.1155/crp/5582480

**Published:** 2026-03-11

**Authors:** Xiaoyu Chen, Cheng Tong, Jie Wang, Yue Wu, Tuo Feng, Jing Wang, Zedong Gong, Yingzhi Chen, Shiyong Chen, Xiaoming Jin, Sisi Chen, Zhongxiu Guo, Xuan Chen, Zeming Ren, Guanhai Dai, Yeling Tong, Xiyu Mei, Renzhao Wu, Xiaomin Xue

**Affiliations:** ^1^ School of Basic Medical Sciences, Zhejiang Chinese Medical University, Hangzhou, China, zcmu.edu.cn; ^2^ Zhejiang Academy of Traditional Chinese Medicine, Hangzhou, China, zcmu.edu.cn; ^3^ Linping Campus, The Second Affiliated Hospital of Zhejiang University School of Medicine, Hangzhou, China, z2hospital.com; ^4^ The First School of Clinical Medicine, Zhejiang Chinese Medical University, Hangzhou, China, zcmu.edu.cn; ^5^ Stark Neuroscience Research Institute and Department of Anatomy, Cell Biology and Physiology, Indiana University School of Medicine, Indianapolis, Indiana, USA, indiana.edu; ^6^ Zhejiang Provincial Key Laboratory of Traditional Chinese Medicine for Pharmacodynamic Material Basis Research of Chinese Medicine, Hangzhou, China

**Keywords:** angiotensin, angiotensin II typeII receptors, compound dendrobium candidum, refractory hypertension, spontaneously hypertensive rat

## Abstract

**Background:**

Compound dendrobium candidum (CDC) is formulated from *Dendrobium candidum* and fragrant peony. Preliminary studies have demonstrated that the combination of CDC with conventional antihypertensive medications exhibits significant synergistic effects in lowering blood pressure. The objective of this study was to evaluate the synergistic effect of combining CDC with antihypertensive medications on refractory hypertension in spontaneously hypertensive rats (SHRs) and to elucidate the underlying mechanisms involved.

**Methods:**

SHRs were treated with either CDC alone or CDC combined with two or three antihypertensive agents including irbesartan, amlodipine, or terazosin, for a duration of 6 weeks. The alterations in blood pressure, angiotensin II (Ang II), insulin, blood sugar, angiotensin II type I receptor (AT_1_R), angiotensin II type II receptor (AT_2_R), insulin receptor, insulin *α* receptor, and insulin β receptor levels were assessed. Glomerular endothelial cells from refractory SHR were then taken for overexpression and knockdown of AT_2_R gene and co‐cultured with CDC serum to measure the expression levels of Ang II receptor gene and protein.

**Results:**

In comparison to the findings observed in the irbesartan + amlodipine + terazosin (IAT) group, the addition of CDC significantly enhanced antihypertensive efficacy. The rate of achieving blood pressure targets (< 150 mmHg) in SHRs with refractory hypertension increased from 0% to 100%. Treatment with CDC significantly reduced the compensatory increase in AT_1_R and AT_2_R levels caused by IAT treatment and showed a significant antihypertensive and synergistic effect. Primary glomerular endothelial cells extracted from SHRs and Wistar rats and treated with 0.5% CDC‐containing serum showed significantly reduced AT_2_R levels in the AT_2_R‐overexpression condition. The combination of CDC and antihypertensive drugs was effective in reducing the messenger RNA (mRNA) and protein expression levels of AT_1_R and AT_2_R in glomerular endothelial cells.

**Conclusions:**

CDC in combination with antihypertensive drugs showed a synergistic effect in controlling refractory hypertension. The mechanism of action may be related to the attenuation of excessive expression of AT_2_R. This study offers a novel approach for the treatment of clinically resistant hypertension.

## 1. Introduction

Hypertension is the most common chronic disease globally and serves as a significant risk factor for disability and premature mortality, resulting in > 9 million deaths each year [1]. Current pharmacological treatment for hypertension consists solely of single compounds that target specific sites of blood pressure regulation at normal levels. The management of drug‐induced hypotension has been employed to provide symptomatic relief from hypertension symptoms. However, prolonged treatment with such medications may lead to a conflict between the effects of antihypertensive drugs and the body’s compensatory mechanisms, thereby increasing the risk of tumors development [2]. In contrast, traditional Chinese medicine (TCM) formulations employ a diverse of compounds that act on multiple targets to regulate blood pressure effectively. Refractory hypertension (RH) is defined by an inability to achieve the target systolic blood pressure (SBP) despite treatment with three different classes of antihypertensive drugs (including diuretics) or necessitating four or more antihypertensive medications to attain SBP < 140 mmHg and/or diastolic blood pressure (DBP) < 90 mmHg [3, 4]. Approximately 46% of American adults suffer from hypertension, with RH accounting for 16% of all patients with hypertension [5]. Given that RH is primarily managed through pharmacological interventions [3], it frequently presents a range of effects that significantly increase the risk of mortality, kidney disease, myocardial ischemia, congestive heart failure, and cerebrovascular events [6]. A survey identified 24 distinct adverse effects associated with antihypertensive medications, with an incidence rate of 85% and a patient treatment noncompliance rate of 34.5% [7]. Due to the common practice of using multiple antihypertensive agents concurrently over extended periods, these adverse reactions are prominent and reduce patient compliance, which is an important factor in the occurrence of RH. Therefore, there is an urgent need to enhance both the efficacy and safety profiles of antihypertensive therapies. Recent studies have reported that the combined use of TCM and antihypertensive drugs can increase the antihypertensive effect [8]. Our preliminary research has suggested that compound dendrobium candidum (CDC), which consists of *Dendrobium candidum* and fragrant peony, exhibits a favorable dose‐ and efficacy‐dependent relationship in spontaneously hypertensive rats (SHRs). The combination of CDC with irbesartan and amlodipine has been shown to enhance blood pressure reduction, probably through mechanisms related to the angiotensin II type I receptor (AT_1_R), angiotensin II type II receptor (AT_2_R), and insulin receptor (ISR) [9]; these effects may also be related to insulin resistance (IR) [10, 11]. Notably, none of the existing antihypertensive drugs function by regulating the expression of AT_2_R.

To further enhance the efficacy of antihypertensive treatment, our research team conducted preliminary experiments in which CDC was combined with hydrochlorothiazide (a diuretic), irbesartan (angiotensin receptor blocker [ARB]), amlodipine (calcium channel blocker [CCB]), enalapril (angiotensin‐converting enzyme inhibitor [ACEI]), and terazosin (*α*1 receptor blocker). The combination of CDC with an ARB and CCB yielded relatively superior results in terms of both the degree of blood pressure reduction and 24‐h blood pressure stability. Moreover, the addition of terazosin further augmented the antihypertensive effect. Therefore, we have established a treatment regimen involving the concurrent use of CDC with CCB, ARB, and *α*1 receptor blockers.

In comparison with single antihypertensive drugs, combinations of two antihypertensive agents did not demonstrate a significant improvement in antihypertensive efficacy [12, 13]. In contrast to the findings in Wistar rats, activation of AT_2_R in SHRs has been reported to induce vasoconstriction. This finding contradicts the prevailing academic view that “AT_2_R promotes vasodilation” [14]. Notably, this finding aligns with the results presented in this study. In this study, the antihypertensive effect in the IA + CDC group was better than that in the IA group, and the effect in the IA + CDC group was better than that in the IAT group at 24‐h postgavage. Furthermore, the relative messenger RNA (mRNA) expression levels of AT_1_R and AT_2_R greatly increased when two antihypertensive drugs are used, which may explain why no synergistic effect was observed with multiple antihypertensive drugs. In this paper, under the premise of achieving the abovementioned excellent antihypertensive effect, some antihypertensive mechanisms are also studied.

TCM formulations employ multicomponent and multitarget treatment methods. Thus, in comparison with antihypertensive drugs, CDC shows complex multitarget and multichannel interactions simultaneously. However, the antihypertensive effects of such compound formulations can only be studied step‐by‐step with multiple single‐component and single‐target studies. In this paper, only two targets, AT_1_R and AT_2_R, were primarily studied.

Therefore, this study aimed to determine the synergistic effect of the combination of CDC and various antihypertensive drugs and the related mechanisms in terms of AT_1_R and AT_2_R expression, providing a new scheme for the treatment of RH.

## 2. Methods

### 2.1. Experimental Animals

All animal experiments were conducted in accordance with the ARRIVE 2.0 reporting guidelines and were approved by the Laboratory Animal Welfare Ethics Committee of Zhejiang Academy of Traditional Chinese Medicine (Approval No.: Zhejiang Zhongyan Animal Ethics ShenziNo. [2022]054). The animals were housed in a specific pathogen‐free (SPF) barrier environment under controlled conditions: temperature 22 ± 2°C, humidity 50 ± 10%, and a 12/12‐h light/dark cycle. Each cage housed no more than four rats, with free access to standard irradiated feed and sterile drinking water; bedding was changed twice weekly. The primary endpoint of this study was the change in SBP after 6 weeks of treatment, and secondary endpoints included serum biochemical indicators and molecular expression levels in renal tissues. All surgical and blood collection procedures were performed under isoflurane inhalation anesthesia. At the experimental endpoint, euthanasia was performed by cervical dislocation following blood collection. Throughout the study, animals were monitored daily, and no signs of abnormal distress were observed.

Forty‐nine 6‐month‐old male SHRs (weight: 321.53 ± 24.92 g [details provided in Supporting Table 1]) were purchased from Beijing Vital River Laboratory Animal Technology Co., Ltd. (certificate number: 110011220106686012). Eight male 6‐month‐old Wistar rats (weight: 570.38 ± 51.83 g) were purchased from the Tongxiang Branch of Zhejiang Vital River Laboratory Animal Technology Co., Ltd. (certificate number: 20220706Aaa0600999941). To establish a model of refractory hypertension (RH), SHRs were administered a triple‐drug regimen (irbesartan + amlodipine + hydrochlorothiazide [IAH]) for 4 weeks. SHRs with SBP persistently > 150 mmHg after this screening period were considered RH and were then randomly allocated into the experimental groups detailed in Table 1 (*n* = 8 per group). The selection of terazosin over hydrochlorothiazide for the triple‐drug base regimen (IAH) was based on preliminary experiments and clinical rationale. Alpha‐1 blockers, such as terazosin, are recommended as an add‐on therapeutic option for RH due to their distinct mechanism of action when conventional triple therapy fails [15, 16]. Our preliminary experiments indicated a more pronounced synergistic antihypertensive effect when CDC was combined with the terazosin‐containing (versus hydrochlorothiazide‐containing) triple‐drug combination. Age‐matched Wistar rats served as the normotensive control group (normal group).

**TABLE 1:** Experimental groups and interventions.

Group name	Animal type	Screening phase medication (4 weeks)	Treatment phase medication (6 weeks)	Description
Normal	Wistar rats	Drinking water	Drinking water	Healthy control
Model	SHRs with RH	IAH	Drinking water	Disease model control
CDC	SHRs with RH	IAH	CDC	Herbal medicine control
IA	SHRs with RH	IAH	Irbesartan + amlodipine	Dual‐drug control
IA + CDC	SHRs with RH	IAH	Irbesartan + amlodipine + CDC	Dual‐drug + CDC
IAT	SHRs with RH	IAH	Irbesartan + amlodipine + terazosin	Triple‐drug control
IAT + CDC	SHRs with RH	IAH	Irbesartan + amlodipine + terazosin + CDC	Triple‐drug + CDC

*Note:* IAH = irbesartan + amlodipine + hydrochlorothiazide, used for screening SHRs with refractory hypertension (RH). All administrations were performed via intragastric gavage.

### 2.2. Pharmaceutical Preparations

CDC consists of the dried stem of *Dendrobium officinale* Kimura et Migo (family Orchidaceae) and the dried root of *Paeonia lactiflora* Pall. (family Paeoniaceae) in a fixed 4:3 ratio. All herbal materials complied with the relevant standards of the Chinese Pharmacopoeia (2020 edition). CDC chewable tablets (specification: 1.5 g/tablet) were provided by the Zhejiang Provincial Academy of TCM (batch number: 201905002). The tablets contain approximately 0.777 g of total herbal material per gram and were powdered for administration. The CDC solution was prepared by adding 13.51 g of powder to 100 mL of ultrapure water, yielding a final concentration of 0.105 g/mL (total herbal material). The daily dosage of CDC was 2.1 g/kg.

The dosages of conventional drugs were irbesartan (batch number: 19067311; Hanhui Pharmaceutical Co., Ltd.) 15 mg/kg/day; amlodipine (batch number: 191251304; Suzhou Dongrui Pharmaceutical Co., Ltd.) 0.5 mg/kg/day; terazosin (batch number: 20050412; Jiangsu Pharmaceutical Co., Ltd.) 0.2 mg/kg/day; and hydrochlorothiazide (batch number: D211105; Yunpeng Pharmaceutical Group Co., Ltd.) 2.5 mg/kg/day.

Chemical characterization of the CDC formulation: The chemical profile of the CDC chewable tablets (batch 201905002) used in this study has been characterized in our previously published work using UPLC‐Q‐TOF/MS analysis [17]. The complete dataset, including the representative total ion chromatogram and detailed mass spectra for the identification of major constituents (e.g., kaempferide, rutin, syringaldehyde, paeoniflorin, and hyperoside), is provided as Supporting Figure S1.

Preparation of CDC‐containing serum: Three healthy male Wistar rats received CDC (2.1 g/kg/day) by oral gavage for three consecutive days. Three hours after the final dose, blood was collected under isoflurane anesthesia. After standing at 4°C for 30 min, samples were centrifuged (3000 rpm, 15 min). The serum was aliquoted, inactivated at 56°C for 30 min, and stored at −80°C.

Rationale for the concentration of CDC‐containing serum in cell experiments: The concentration of 0.5% CDC‐containing serum used for in vitro treatments was selected based on preliminary experiments and established methodology in serum pharmacology for TCM [18]. This concentration was found to effectively elicit biological responses in primary glomerular endothelial cells while avoiding nonspecific cytotoxicity or overactivation that can occur with higher serum concentrations, thereby ensuring both the physiological relevance and reproducibility of the observed effects.

Preparation of normal (drug‐free) serum: Serum was prepared from three healthy male Wistar rats administered an equal volume of drinking water, following the same procedure as above.

### 2.3. Blood Pressure Measurement

The rats were kept warm at 38.3°C in a DK‐450B electric constant‐temperature water bath (Shanghai Senxin Experimental Instrument Co., Ltd.), using a multigradient heating method. First, they were heated for 4 min. If a blood pressure measurement could not be obtained, the rats were heated for 2‐3 min again until they could be assessed with a BP‐2010A noninvasive tail artery blood pressure measuring instrument (Nippon Softron Co., Ltd,Japan). We obtained 6–10 blood pressure measurements and averaged them to determine the SBP at 3 and 24 h post‐dose. These time points were selected to capture the peak (3 h) and trough (24 h) antihypertensive effects, which constitutes a standard method for evaluating the consistency of 24‐h blood pressure control (e.g., trough‐to‐peak ratio). This selection was based on preliminary experiments indicating that the maximum blood pressure‐lowering effect consistently occurred approximately 3 h post‐dose under our experimental conditions. These measurements reflected the peak and valley SBP values to assess antihypertensive efficacy. The blood pressure measurements of the rats were obtained during the day, and their diet and drinking water were not restricted.

### 2.4. Blood Analysis

After 6 weeks of treatment, blood was collected from the tail artery of four rats in each group to measure the fasting blood glucose (FBG) level (ACCU‐CHEK, USA). After anesthesia, blood was collected from the abdominal aorta and serum was separated. An angiotensin II (Ang II) enzyme‐linked immunosorbent assay (ELISA) kit (Enzyme‐linked Biotechnology, Shanghai, China) and an insulin (INS) ELISA kit (Enzyme‐linked Biotechnology, Shanghai, China) were used to detect the serum Ang II and INS content. We continued to observe the blood pressure after stopping drug administration in the remaining animals in each group.

### 2.5. Real‐Time Fluorescence Quantitative Polymerase Chain Reaction

Total mRNA was extracted from the renal cortex or glomerular endothelial cells with RNAiso Plus 9109 reagents (Takara, Dalian, China). The RNA concentration was determined by the nanodrop method (Thermo, NANODROP 2000, USA). A PrimeScript RT kit (TaKaRa, Dalian, China) was used to reverse‐transcribe the RNA into complementary DNA (cDNA). Real‐time polymerase chain reaction was then performed on a 7500 StepOnePlus system (Applied Biosystems, Foster, CA, USA). A TB Green Premix Ex TaqTM Series II kit (TaKaRa, Dalian, China) was used to detect the mRNA expression levels of AT_1_R, AT_2_R, ISR, insulin *α* receptor (ISR‐α), insulin β receptor (ISR‐β), and β‐actin. All primers were synthesized by Shanghai Sangon Bioengineering Technology Co., Ltd. (Sangon, Shanghai, China) (details provided in Supporting Table 2). The analysis was performed using the 2^−ΔCt^ method.

### 2.6. Western Blotting

A total protein extraction kit (KeyGEN bioTECH, Jiangsu, China) was used to extract proteins from the renal cortex and renal endothelial cells, and a bicinchoninic acid (BCA) kit (Beyotime, Shanghai, China) was used to determine the protein concentration. The protein‐loading amount for the renal cortex was 10 μg/well and that for the glomerular endothelial cells was 4 μg/well. The proteins underwent 10% sodium dodecyl sulfate‐polyacrylamide gel electrophoresis (SDS‐PAGE), followed by transfer to a polyvinylidene difluoride (PVDF) membrane (Immobilon‐P, USA). The membranes were subsequently blocked with tris‐buffered saline with tween 20 (TBST) solution containing 10% skim milk (Beyotime, China) at room temperature for 1 h. The membranes were then washed four times with TBST, followed by overnight incubation with primary antibodies against GAPDH (1:1000: Diagbio, Hangzhou, China) and AT_1_R and AT_2_R (1:1000: Abcam, Cambridge, MA, USA). The next day, the membranes were washed with TBST and underwent incubation with the secondary antibody (Goat Anti‐Rabbit IgG [*H* + *L*] HRP; 1:100; Affinity, Jiangsu, China) at room temperature for 2 h. Finally, the membranes were washed with TBST followed by incubation in ECL developing solution (Biosharp, China) for 5–8 min. Images were captured using a laser scanner (ImageQuant LAS 500; GE, USA). Adobe Photoshop 2021 was used to analyze the results. The cumulative density value of the target protein was normalized to that of the internal reference protein (GAPDH).

### 2.7. Cell Extraction and Identification

Tissue samples of the renal cortex were cut into small pieces and digested with 0.1% type IV collagenase (STEMCELL Technologies Inc, Vancouver, Canada) at 37°C for 30 min. The digested renal cortex was passed through 100‐, 200‐, and 400‐mesh sieves in sequence to remove excess tubular and cell debris. Only glomeruli were obtained after passing the digested samples through the 400‐mesh sieve. Glomerular endothelial cells were washed and cultured with complete endothelial cell culture medium (Scien Cell Research Laboratories, America) at 37°C and 5% CO_2_. Immunofluorescence staining for von Willebrand factor (VWF) and CD31 was performed to identify endothelial cells. After removing the culture medium, glomerular endothelial cells were washed with phosphate‐buffered saline (PBS) three times on a shaking table at 110 rpm for 5 min and fixed in 4% paraformaldehyde for 15 min. After three more PBS washes, the cells were perforated with 0.1% Triton X‐100 for 10 min, washed with PBS three times, and blocked for 1 h in the blocking solution. The cells were incubated with primary antibodies against VWF and CD31 (1:200; Affinity, Jiangsu, China) overnight at 4°C on the shaker. The next day, the cells were washed three times in PBS followed by incubation with the secondary antibody (1:100; EarthOX, LLC, San Francisco, CA, USA) in the dark for 2 h. The cells were then washed in PBS three times, and 4′,6‐diamidino‐2‐phenylindole (DAPI; 1:1000 BOSTER, Wuhan, China) was added for 5 min to stain the nuclei, which was followed by three washes and drying. Finally, a drop of fluorescence quenching solution (Vector Laboratories, USA) was added, and the glass slide was sealed. Images were obtained with a fluorescent microscope (TE 2000‐U microscope; Nikon Eclipse, Japan).

### 2.8. Cell Transfection and Group Therapy

Glomerular endothelial cells from SHRs were infected with lentiviruses (Genepharma, Shanghai, China) that knocked down or overexpressed AT_2_R genes or did not affect AT_2_R gene expression to create the AT_2_R knockdown group (AT_2_R K group), AT_2_R‐overexpression group (AT_2_R O group), AT_2_R knockdown negative control group (AT_2_R kN group), and AT_2_R‐overexpression negative control group (AT_2_R ON group). The transfection status was imaged under a fluorescence microscope. The cells were treated with 0.5% CDC‐containing serum (obtained from Wistar rats after 3 days of CDC administration and inactivated at 56°C for 30 min) or 0.5% drug‐free serum (obtained from Wistar rats treated with drinking water for 3 days).

### 2.9. Statistical Analysis

**FIGURE 1:**
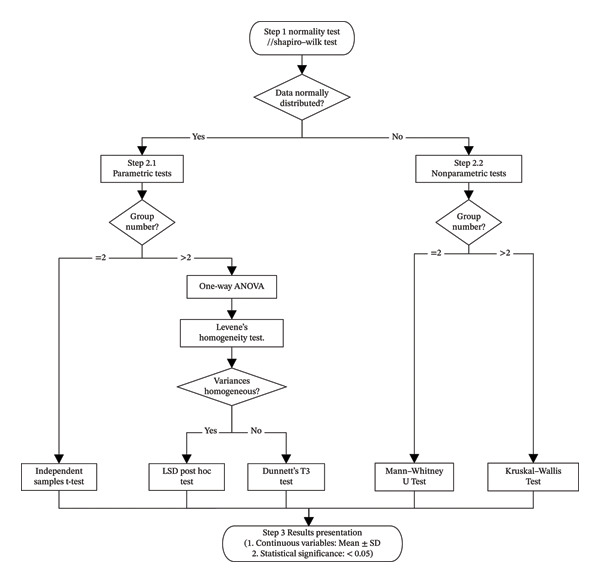
Flowchart of statistical analysis.

All data were analyzed using SPSS 25.0 software (IBM Corp., Armonk, NY, USA). The detailed statistical analysis procedure is outlined in the flowchart presented in Figure 1. In brief, the normality of distribution was assessed using the Shapiro–Wilk test. Based on the distribution and variance homogeneity (evaluated by Levene’s test), appropriate parametric (one‐way ANOVA with post hoc tests or independent samples *t*‐test) or nonparametric (Kruskal–Wallis or Mann–Whitney *U* test) methods were applied. Continuous data are presented as the mean ± standard deviation (*x* ± *s*). A *p* value < 0.05 was considered statistically significant.

## 3. Results

### 3.1. Animal Experiments

#### 3.1.1. SBP During the Screening Period for SHRs With RH

After 4 weeks of treatment with three antihypertensive drugs, including diuretics, the blood pressure of all elderly SHRs with RH failed to reach the standard value. SHRs were considered to be hypertensive once their SBP was > 150 mmHg [19]. SHRs are widely used models in studies of hypertension due to their genetic similarity to human essential hypertension [20]. In accordance with previous reports [21, 22], SHRs were treated with antihypertensive drugs for 4 weeks to identify SHRs with RH. Before receiving drug treatment in the RH screening period, the SBP of the SHRs was significantly higher than that in the normal group (*p* < 0.01) (Figure 2(a)). The SBP of SHRs 3 h post‐dose after 4 weeks of screening treatment was > 150 mmHg, and that at 24 h post‐dose after 4 weeks of gavage administration was still > 150 mmHg. Therefore, these SHRs could be considered to have RH for the treatment study (details provided in Supporting Table 3).

FIGURE 2SBP changes across groups. (a) Effect of the combined use of three antihypertensive drugs (containing hydrochlorothiazide) on SBP during the screening period of SHRs (normal: *n* = 8, model *n* = 49; comparison between the normal and model groups:  *p* < 0.01). (b) SBP changes across groups before and after treatment (except for 3 days after drug withdrawal, *n* = 4, and *n* = 8 in each group in other periods). Note: comparisons between each treatment group and model groups:  *p* < 0.01; comparisons between the IA, IAT, IA + CDC, IAT + CDC and CDC groups:  *p* < 0.01, ; comparison between the IA + CDC and IA groups: 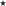 *p* < 0.01, 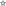 *p* < 0.05; comparison between the IA + CDC, IAT + CDC, and IAT groups: 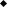 *p* < 0.01, 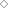 *p* < 0.05; comparison between the IAT + CDC and IA + CDC groups: 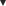 *p* < 0.01. (c) SBP reduction in each group after treatment (except for 3 days after drug withdrawal, *n* = 4, and *n* = 8 in each group in other periods). Note: comparisons between each treatment group and model groups:  *p* < 0.01,  *p* < 0.05; comparisons between the IA, IAT, IA + CDC, IAT + CDC, and CDC groups:  *p* < 0.01,  *p* < 0.05; comparison between the IA and IA + CDC groups: 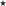 *p* < 0.01, 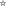 *p* < 0.05; comparisons between the IA + CDC, IAT + CDC, and IAT groups: 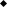 *p* < 0.01, 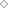 *p* < 0.05; comparisons between the IAT + CDC and IA + CDC groups: 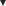 *p* < 0.01, 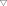 *p* < 0.05.

**Figure:**
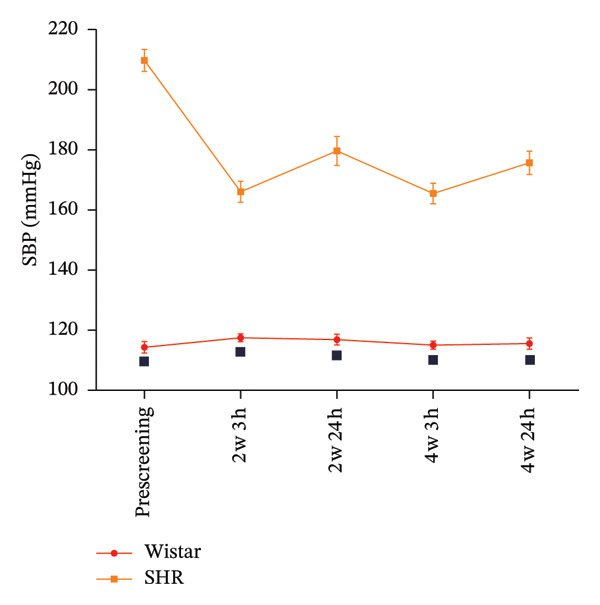
(a)

**Figure:**
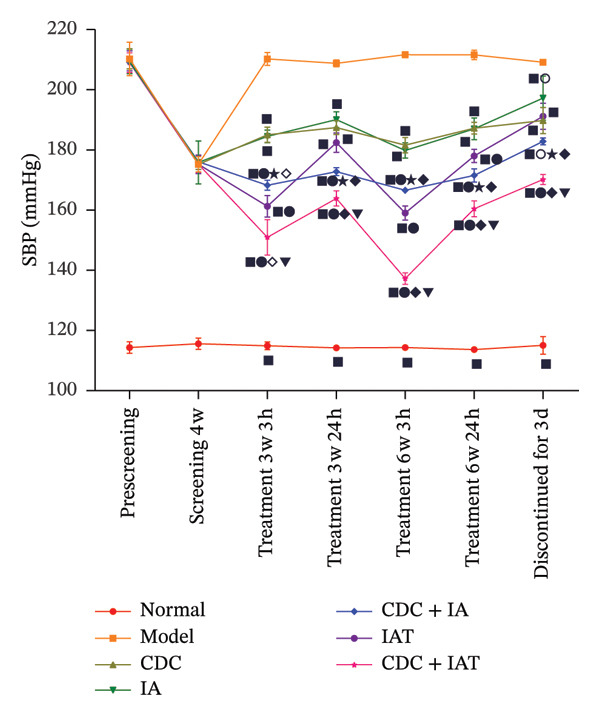
(b)

**Figure:**
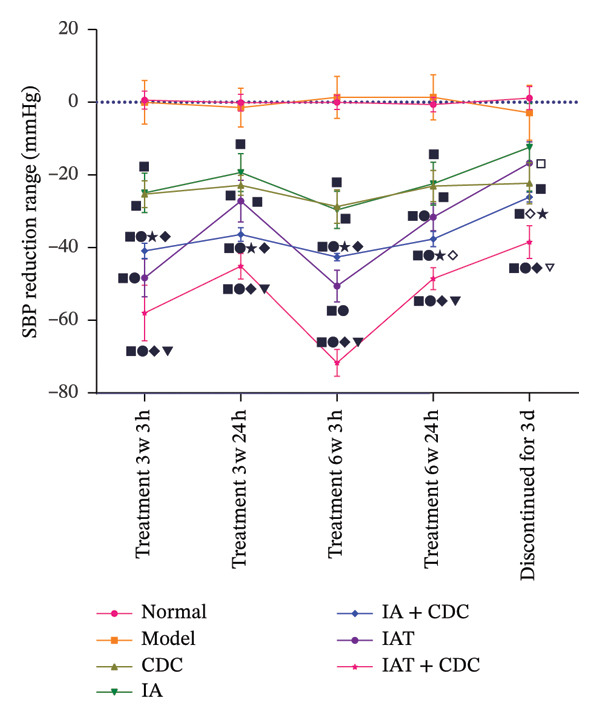
(c)

#### 3.1.2. SBP in SHRs With RH: The Blood Pressure Reduction and the Stability of the Blood Pressure–Reducing Effect in the IA + CDC Group Were Higher Than Those in the IAT Group

The SBPs in all treatment groups were significantly lower than that in the model group (*p* < 0.01). After 6 weeks of treatment, the SBP in the IAT group was significantly lower than that in the IA + CDC group when measurements were obtained 3 h post‐dose (*p* < 0.01); however, the measurements obtained 24 h post‐dose in the IAT group were significantly higher than those in the IA + CDC group (*p* < 0.01).

After 6 weeks of treatment, while the IA + CDC group showed a lower maximum blood pressure–lowering amplitude than the IAT group at 3 h post‐dose, at 24 h post‐dose, it showed a better antihypertensive effect than the IA and IAT groups, reduced blood pressure variability, and a more effective and stable blood pressure–lowering effect. Blood pressure fluctuations in the IA + CDC group at 3–24 h post‐dose after 6 weeks of treatment were significantly lower than those in the IAT group (*p* < 0.01). A comprehensive evaluation of antihypertensive amplitude and antihypertensive stability at 24 h post‐dose showed high blood pressure fluctuations in the IAT group and an antihypertensive effect that was significantly lower than that in the IA + CDC group (*p* < 0.01). After 3 days of drug withdrawal, the SBP in the IA + CDC group was significantly lower than those in the IA and IAT groups (*p* < 0.01). Thus, the persistence of the antihypertensive effect in the IA + CDC group was better than those in the IA and IAT groups.

The 24 h post‐dose SBP in the CDC group was significantly lower than that in the model group (*p* < 0.01) at 6 weeks of treatment; at the same time, the CDC group showed a similar antihypertensive range as the IA group. After stopping treatment for 3 days, the SBP in the CDC group was significantly lower than those in the model and IA groups (*p* < 0.01); thus, the CDC group showed a more lasting antihypertensive effect than the IA group (Figures 2(b), 2(c); details provided in Supporting Table 4).

#### 3.1.3. SBP Compliance Rate: The Combination of the Three Antihypertensive Drugs and CDC Significantly Improved the SBP Compliance Rate

After 6 weeks of treatment, only the IAT + CDC group showed a 100% SBP compliance rate for SBP < 140 mmHg at 3 h post‐dose, which significantly differed from the corresponding findings for the IA + CDC and IAT groups (*p* < 0.01). Furthermore, the compliance rate for SBP < 160 mmHg in the IAT + CDC group was 37.5% higher than that in the IAT group. At 24 h post‐dose after 6 weeks of treatment, the IAT + CDC group still showed a 50% compliance rate for SBP < 160 mmHg; the corresponding compliance rate for the other treatment groups was 0% (Table 2).

**TABLE 2:** Effect of the combination of CDC combined with antihypertensive drugs on the SBP compliance rate in the antihypertensive treatment of SHRs with RH for 6 weeks (*n* = 8 per group).

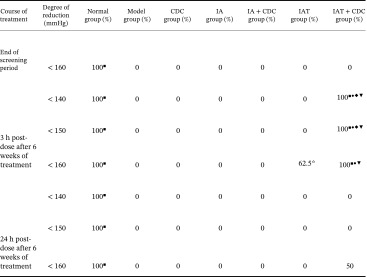

*Note:* Comparisons between each treatment group and the model group:  *p* < 0.01; comparison between the IAT + CDC group and CDC groups:  *p* < 0.01; comparison between the IAT and IA groups: 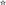 *p* < 0.05; comparison between the IAT + CDC and IAT groups: 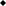 *p* < 0.01; comparison between the IAT + CDC group and IA + CDC groups: 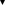 *p* < 0.01.

#### 3.1.4. The Serum Ang II in SHRs With RH: No Significant Change was Observed Between the Treatment Groups and the Model Group

The serum Ang II content in the IA + CDC group showed a certain increase trend compared with the model and IA groups, but there was no statistical significance. The serum Ang II content in the IAT + CDC group was significantly lower than that in the model group (*p* < 0.05) (Figures 3(a)) (details provided in Supporting Table 5).

FIGURE 3The serum Ang II content; the relative expression levels of AT_1_R and AT_2_R mRNA in each group after 6 weeks of treatment (*n* = 4). (a) Ang II content in each group after 6 weeks of treatment (*n* = 4). (b) Relative mRNA expression of AT_1_R in the renal cortex of each group after 6 weeks of treatment (*n* = 4). (c) Relative mRNA expression of AT_2_R in the renal cortex of each group after 6 weeks of treatment (*n* = 4).

**Figure:**
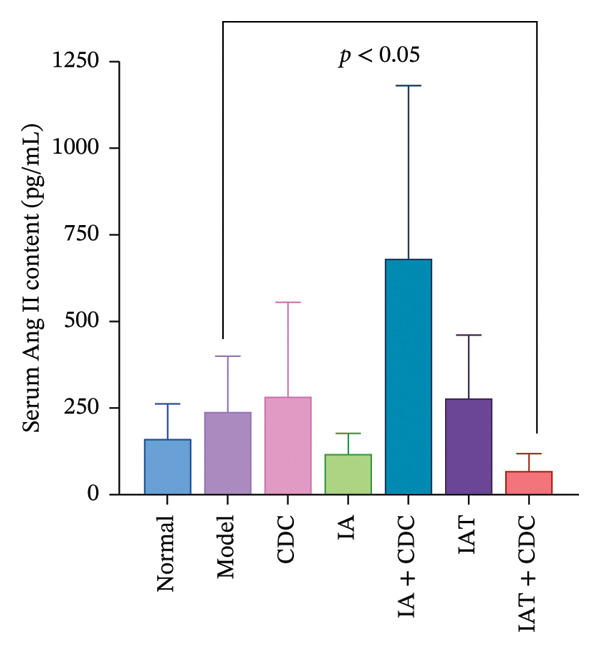
(a)

**Figure:**
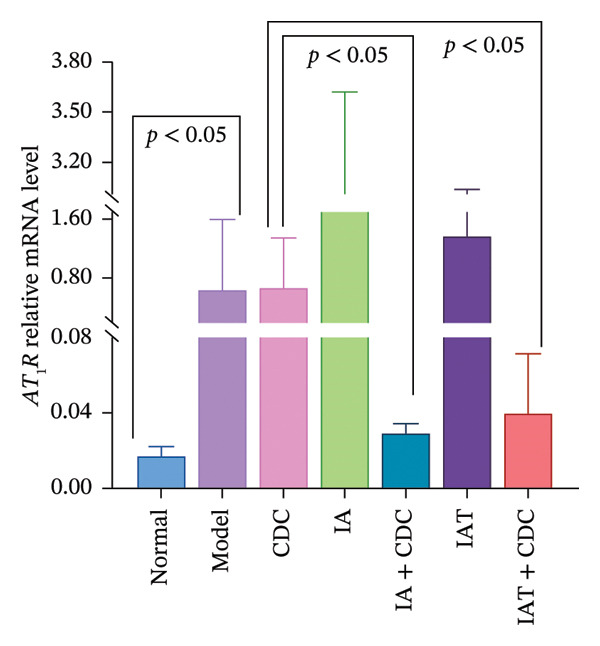
(b)

**Figure:**
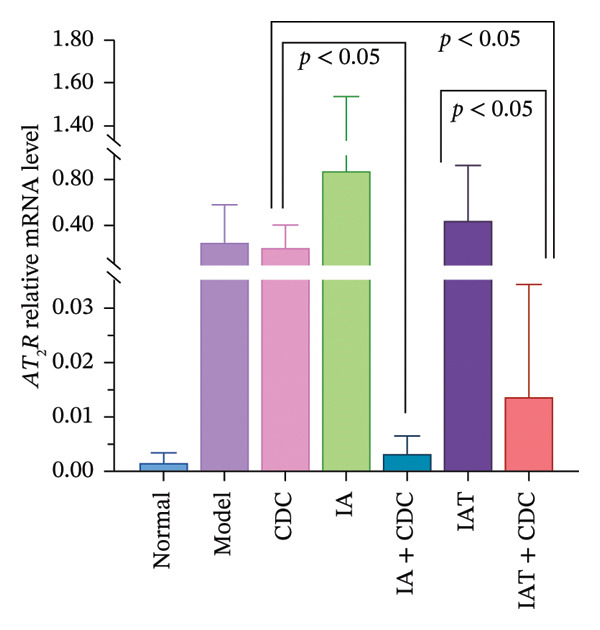
(c)

#### 3.1.5. mRNA Expression Levels of AT_1_R and AT_2_R in the Renal Cortex: The Relative Expression Level of AT_2_R mRNA in the IAT + CDC Group was Significantly Lower Than That in the IAT Group

The relative mRNA expression levels of AT_1_R and AT_2_R in the model group were higher than those in the normal group. Notably, the relative expression level of AT_1_R mRNA in the model group was significantly greater than that in the normal group (*p* < 0.05). In comparison with the model group, both the IA and IAT groups exhibited a compensatory increase in the relative mRNA expression levels of AT_1_R and AT_2_R following antihypertensive treatment; however, this increase did not reach statistical significance. The relative mRNA expression levels of AT_1_R and AT_2_R decreased in the IA + CDC and IAT + CDC groups in comparison with those in the IA and the IAT groups. Specifically, the relative mRNA expression of AT_2_R in the IAT + CDC group was significantly lower than that in the IAT group (*p* < 0.05; Figures 3(b) and 3(c)). The decrease in the relative mRNA expression of AT_1_R was not statistically significant, which may be due to large individual differences and small sample size (details provided in Supporting Table 6).

#### 3.1.6. Expression of AT_1_R and AT_2_R in the Renal Cortex: After Treatment With IA and CDC, the Relative Expression Level of AT_2_R Protein was Significantly Lower Than That in the IA Group

The relative protein expression levels of AT_1_R and AT_2_R in the renal cortex of the model group were slightly higher than those in the normal group, but without statistical significance. After treatment, the relative protein expression levels of AT_2_R in the CDC group showed a downward trend in comparison with the model group, although the difference did not show statistical significance. The relative protein expression levels of AT_1_R and AT_2_R in the renal cortex of the IA group were slightly higher than those in the model group; however, the IA + CDC group showed significant reductions in the expression levels of both proteins in comparison with the model and IA groups (*p* < 0.05). Furthermore, the IAT + CDC group showed a decreasing trend in the expression levels of the two proteins in comparison with the IAT group, but the difference was not statistically significant (Figures 4(a), 4(b), and 4(c); details provided in Supporting Table 7).

FIGURE 4The relative expression levels of AT_1_R and AT_2_R protein in each group after 6 weeks of treatment (*n* = 4). (a) Proteins bands of AT_1_R, AT_2_R, and GAPDH in the renal cortex of each group after 6 weeks of treatment. (b) Protein expression of AT_1_R in renal cortex of each group after 6 weeks of treatment (*n* = 4). (c) Protein expression of AT_2_R in renal cortex of each group after 6 weeks of treatment (*n* = 4).

**Figure:**
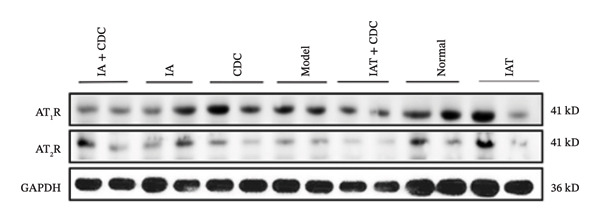
(a)

**Figure:**
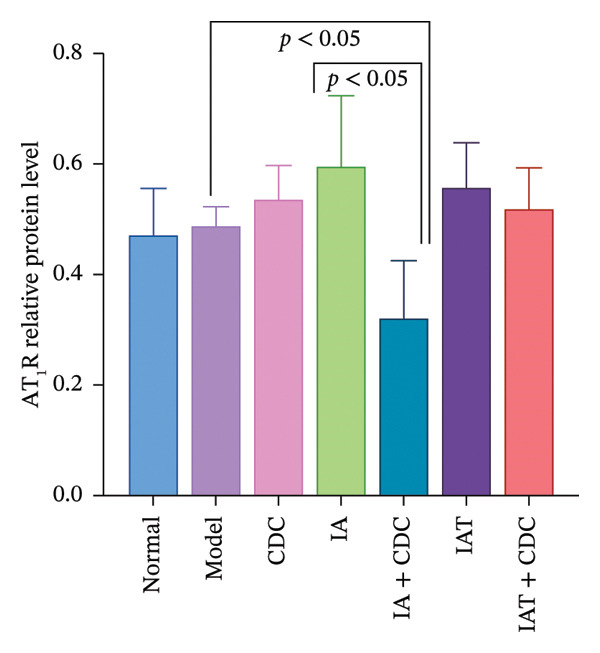
(b)

**Figure:**
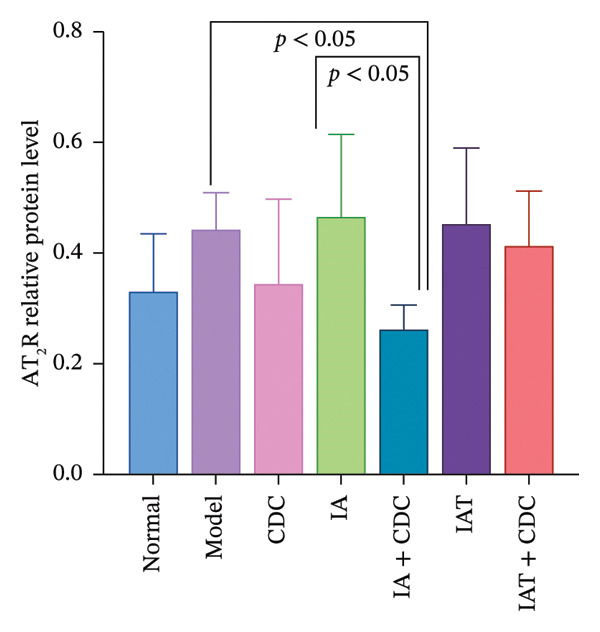
(c)

#### 3.1.7. Serum FBG and INS Levels and the IR Index: The IR Index in the IAT + CDC Group Showed a Significant Reduction

The FBG concentration in the treatment groups did not change significantly in comparison with that in the model group. The serum INS concentration and IR index in each treatment group decreased to some extent in comparison with the model group. The IR index in the IAT + CDC group was significantly lower than that in the model group (*p* < 0.05; Figures 5(a), 5(b), and 5(c)) (details provided in Supporting Table 8).

FIGURE 5Changes in FGB, serum INS concentration, IR, and ISR mRNA expression in each group after treatment. (a) The FBG concentration in each group after 6 weeks of treatment (*n* = 4). (b) The serum INS concentration in each group after 6 weeks of treatment (*n* = 4). (c) The IR index of each group after 6 weeks of treatment (*n* = 4). (d) Relative mRNA expression level of ISR in the renal cortex in each group after 6 weeks of treatment (*n* = 4). (e) Relative mRNA expression level of ISR‐α in the renal cortex in each group after 6 weeks of treatment (*n* = 4). (f) Relative mRNA expression level of ISR‐β in the renal cortex in each group after 6 weeks of treatment (*n* = 4).

**Figure:**
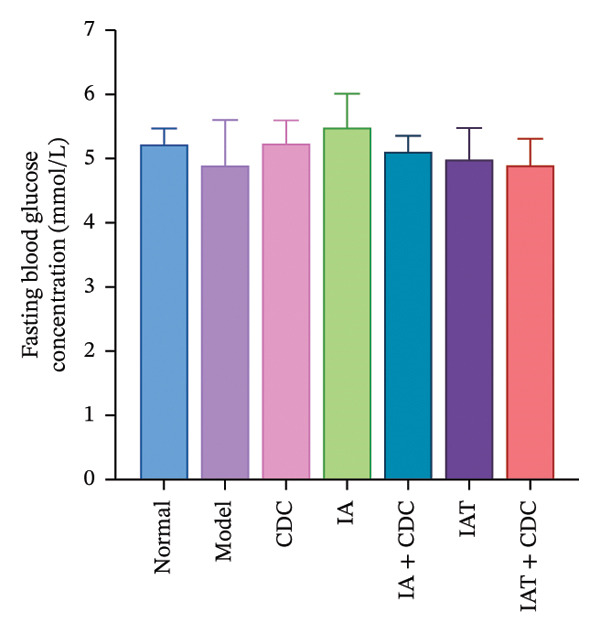
(a)

**Figure:**
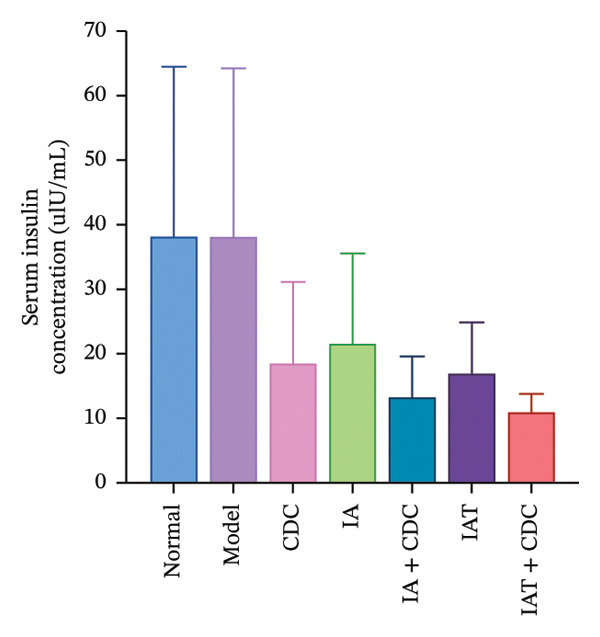
(b)

**Figure:**
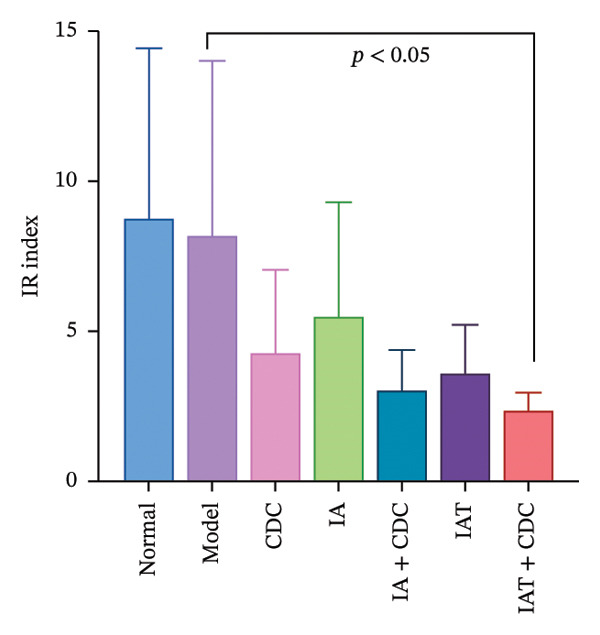
(c)

**Figure:**
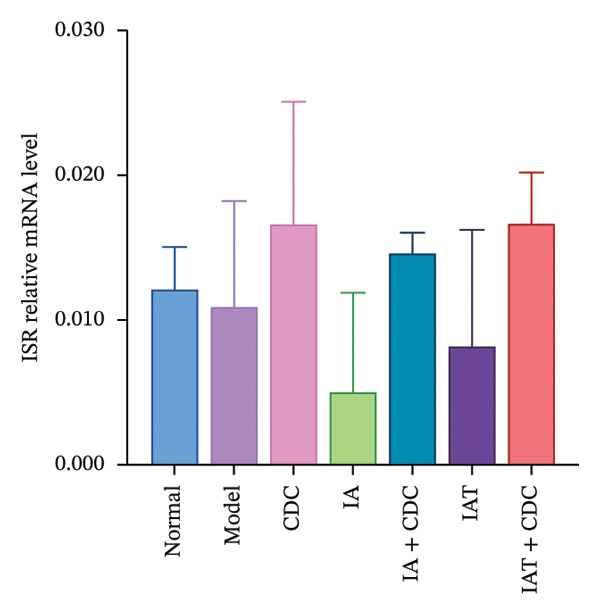
(d)

**Figure:**
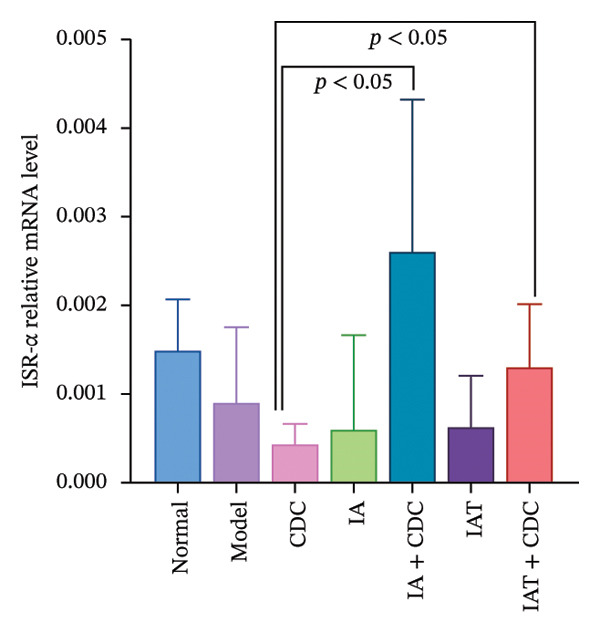
(e)

**Figure:**
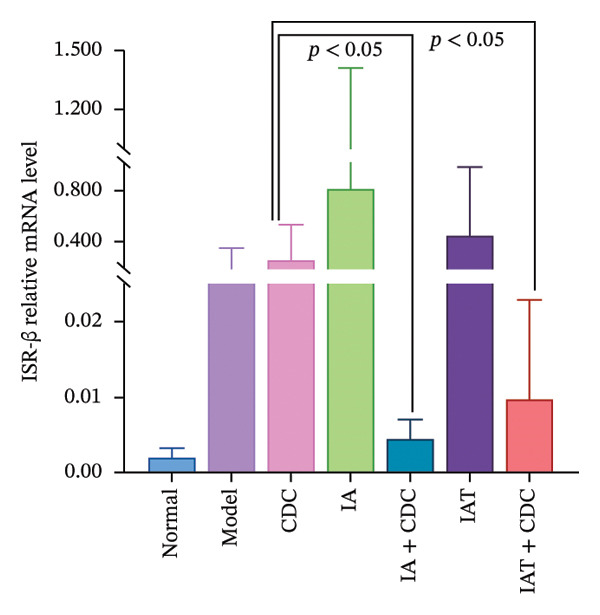
(f)

#### 3.1.8. Relative mRNA Expression Levels of ISR, ISR‐α, and ISR‐β in the Renal Cortex: After Administration of IA and IAT in Conjunction With CDC Treatment, Notable Changes Accompanying the Reduction in Blood Pressure Were Observed

The ISR consists of two subunits, namely, ISR‐α and ISR‐β [23]. In comparison with the model group, the CDC, IA + CDC, and IAT + CDC groups showed an increasing trend in the relative mRNA expression level of ISR. In the IA + CDC and the IAT + CDC groups, ISR‐α expression showed an increasing trend and ISR‐β expression showed a decreasing trend in comparison with the findings in the IA and the IAT groups; however, these trends did not show statistical significance, which may be related to the small sample size (Figures 5(d), 5(e), and 5(f)) (details provided in Supporting Table 8).

### 3.2. Cellular Experiments

#### 3.2.1. Extraction and Identification of Primary Glomerular Endothelial Cells

Spherical glomeruli were extracted from the renal cortex (Figures 6(a)). During incubation at 5% CO_2_ and 37°C, the glomerular endothelial cells wrapped around the glomerulus and crawled outward (Figures 6(b)). Glomerular endothelial cells were detected by immunofluorescence for key endothelial cell markers (VWF and CD31) (Figures 6(c) and 6(d)).

FIGURE 6Primary culture and identification of glomerular endothelial cells from SHR and Wistar rats. (a) Primary glomeruli of SHRs and Wistar rats (magnification, x100) (b). Glomerular endothelial cells of SHRs and Wistar rats after passage (magnification, x100) (c). Glomerular endothelial cells of SHRs and Wistar rats were stained with VWF and DAPI (magnification, x40). (d) Glomerular endothelial cells of SHRs and Wistar rats were stained with CD31 and DAPI (magnification, x40).

**Figure:**
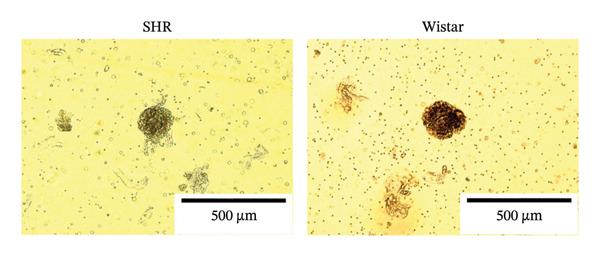
(a)

**Figure:**
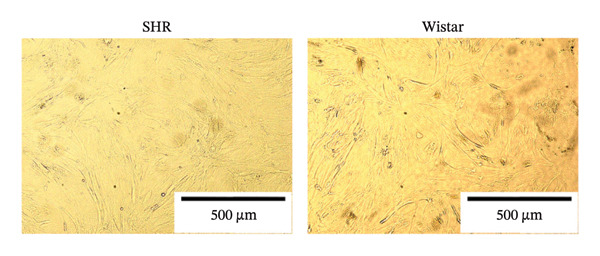
(b)

**Figure:**
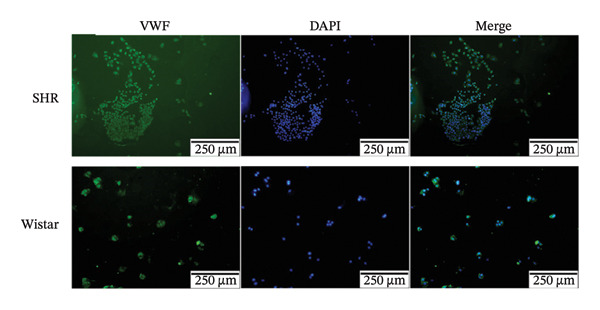
(c)

**Figure:**
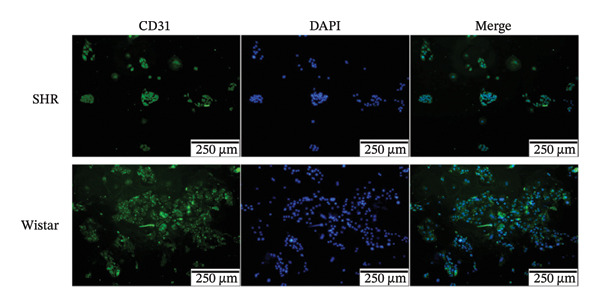
(d)

#### 3.2.2. Transfection of Glomerular Endothelial Cells

**FIGURE 7:**
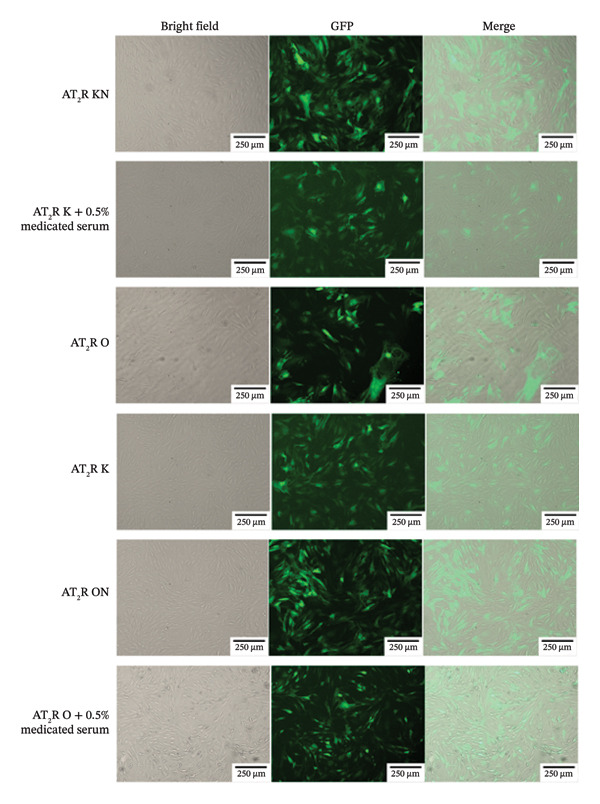
Transfection diagram of glomerular endothelial cells of SHRs (magnification, x100). Note: Transfected virus carries green fluorescent protein, and successful transfection can be seen under fluorescence microscope.

AT_2_R gene knockdown lentivirus particles, AT_2_R gene overexpression lentivirus particles, AT_2_R gene knockdown negative control lentivirus particles, and AT_2_R gene negative control overexpression lentivirus particles were used to infect glomerular endothelial cells isolated from SHRs to create the AT_2_R K, AT_2_R O, AT_2_R kN, and AT_2_R ON groups. Fluorescence imaging was used to identify cells that had been successfully transfected with the virus (Figure 7).

#### 3.2.3. Effect of CDC‐Containing Serum on the Relative mRNA Expression of AT_1_R and AT_2_R in Glomerular Endothelial Cells With AT_2_R Overexpression or Knockdown

Treatment with CDC‐containing serum tended to decrease the relative AT_2_R mRNA expression in the AT_2_R O group but had no effect on the relative AT_2_R mRNA expression in the AT_2_R K group.

The relative mRNA expression levels of AT_1_R and AT_2_R in the model group were significantly higher than those in the normal group (*p* < 0.05). The relative mRNA expression of AT_2_R in the AT_2_R K group was significantly lower than that in the AT_2_R kN group (*p* < 0.05). In comparison with the AT_2_R K group, the AT_2_R *K* + 0.5% CDC‐containing serum group showed an upward trend in the relative mRNA expression levels of AT_1_R and AT_2_R, but without statistical significance. The relative mRNA expression of AT_2_R in the AT_2_R O group was significantly higher than that in the AT_2_R ON group (*p* < 0.05). In comparison with the AT_2_R O group, the AT_2_R *O* + 0.5% CDC‐containing serum group showed a downward trend in relative mRNA expression levels of AT_1_R and AT_2_R, but without statistical significance (Figure 8; details provided in Supporting Table 10).

FIGURE 8The relative expression of AT_1_R and AT_2_R mRNA in glomerular endothelial cells of each group. (a) Effect of CDC‐containing serum and lentivirus transfection on the relative mRNA expression of AT_1_R in glomerular endothelial cells on day 5 of culture (*n* = 4). (b) Effect of CDC‐containing serum and lentivirus transfection on the relative mRNA expression of AT_2_R in glomerular endothelial cells on Day 5 of culture (*n* = 4).

**Figure:**
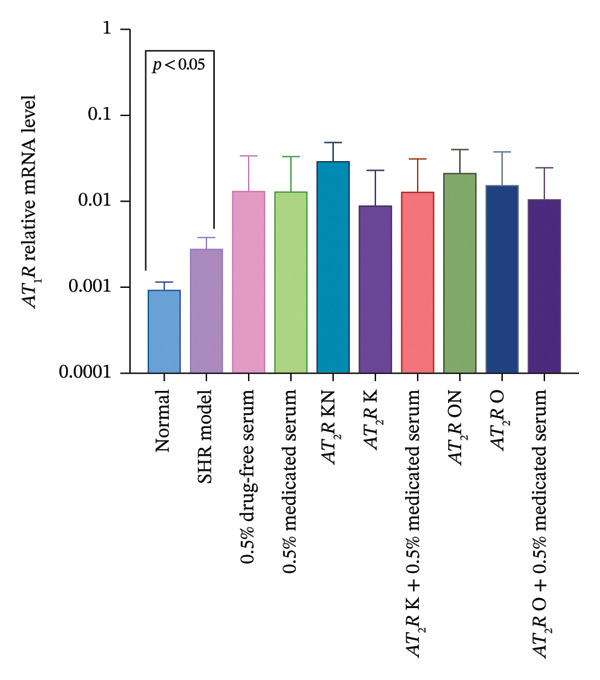
(a)

**Figure:**
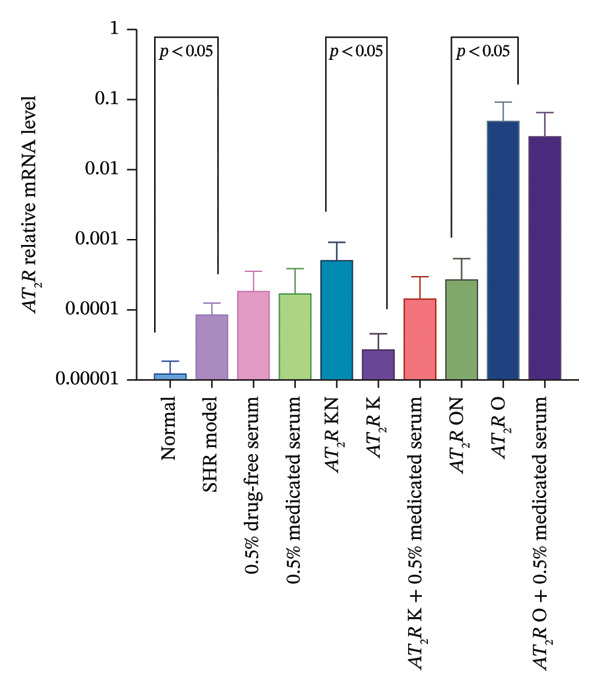
(b)

#### 3.2.4. Effect of CDC‐Containing Serum on the Relative Protein Expression of AT_1_R and AT_2_R in Glomerular Endothelial Cells With AT_2_R Overexpression or Knockdown: The Relative Protein Expression of Overexpressed AT_2_R was Significantly Reduced, But it Had no Effect on the Relative Protein Expression of Knockdown AT_2_R

The relative protein expression levels of AT_1_R and AT_2_R in the model group were significantly higher than those in the normal group (*p* < 0.05). The relative expression levels of AT_1_R and AT_2_R in the AT_2_R K group were significantly lower than those in the AT_2_R kN group (*p* < 0.05). The expression levels of the two proteins showed no statistically significant differences between the AT_2_R *K* + 0.5% CDC‐containing serum group and the AT_2_R K group. Furthermore, AT_2_R expression in the AT_2_R O group was significantly higher than that in the AT_2_R ON group (*p* < 0.01). The relative expression level of AT_2_R in the AT_2_R *O* + 0.5% CDC‐containing serum group was significantly lower than that in the AT_2_R O group (*p* < 0.01). In the model group, treatment with 0.5% CDC‐containing serum or drug‐free serum had no significant effect on the relative expression levels of AT_1_R and AT_2_R; however, the CDC‐containing serum had a significant callback effect on the protein expression of AT_2_R in glomerular endothelial cells after AT_2_R overexpression (*p* < 0.01). The relative expression level of AT_1_R showed a callback trend, but it was not significant. The relative expression level of AT_1_R and AT_2_R in glomerular endothelial cells with AT_2_R knockdown showed no significant recovery (Figure 9; details provided in Supporting Table 11).

FIGURE 9The relative expression of AT_1_R and AT_2_R protein in glomerular endothelial cells of each group. (a) GAPDH, AT_1_R, and AT_2_R protein bands of the transfected and treated glomerular endothelial cells on Day 5 of culture. (b) Effect of CDC‐containing serum and lentivirus transfection on the relative protein expression of AT_1_R in glomerular endothelial cells on Day 5 of culture (*n* = 3). (c) Effect of CDC‐containing serum and lentivirus transfection on the relative protein expression of AT_2_R in glomerular endothelial cells on Day 5 of culture (*n* = 3).

**Figure:**
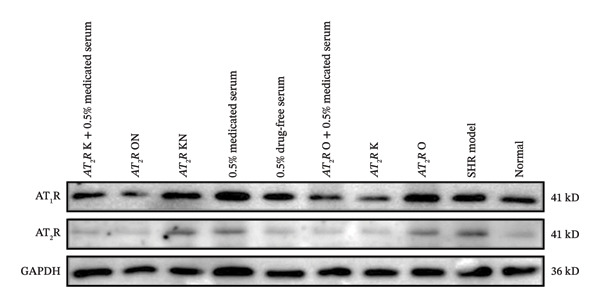
(a)

**Figure:**
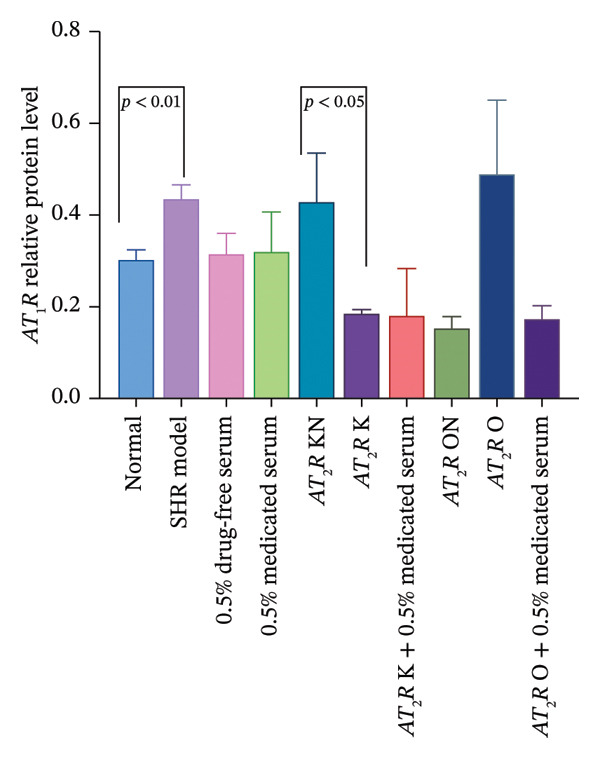
(b)

**Figure:**
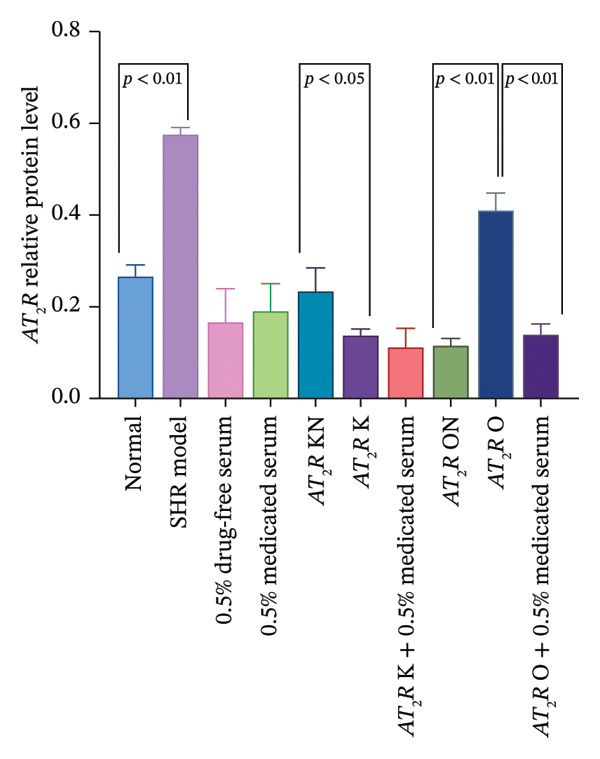
(c)

## 4. Discussion

### 4.1. Main Findings: The Combination of CDC With Conventional Antihypertensive Drugs Synergistically Enhances Efficacy and Suggests Improved Blood Pressure Stability

This study demonstrates that in a RH rat model, CDC combined with conventional antihypertensive regimens exerts significant synergistic effects. This synergy is evident not only in the magnitude of blood pressure reduction but also in the potential improvement of blood pressure stability. Specifically, the CDC‐plus‐IA regimen produced a greater blood pressure–lowering effect than the IAT regimen alone and provided more stable control over 24 h.

Blood pressure fluctuations are an important independent predictor of cardiovascular risk and reflect the quality of blood pressure control [24]. In this study, the CDC‐plus‐IA regimen was associated with reduced blood pressure fluctuation (inferred from the difference between 3‐h and 24‐h post‐dose measurements) compared to IA or IAT alone. This suggests that CDC may improve the stability of the antihypertensive effect conferred by conventional drugs. If confirmed by continuous monitoring, such enhanced stability could be clinically relevant for mitigating target organ damage. Furthermore, the addition of CDC to the IAT regimen increased the peak achievement rate for SBP < 140 mmHg to 100%, substantially improving the therapeutic success rate. To elucidate the mechanism underlying this synergy, we investigated key molecular targets within the renin–angiotensin system.

### 4.2. Exploration of the Core Mechanism: Restoring System Balance by Modulating Renin–Angiotensin–Aldosterone system (RAAS) Receptor Expression, Particularly AT_2_R

At the molecular level, our results indicate a potential mechanism for CDC’s synergistic action. Although conventional regimens (IA or IAT) effectively lowered blood pressure, they concomitantly triggered a further upregulation of both AT_1_R and AT_2_R expression in the renal cortex. Cotreatment with CDC significantly reversed this trend, notably suppressing the abnormal elevation of AT_2_R. This drug‐induced receptor upregulation likely represents a counter‐regulatory feedback mechanism against therapeutic blood pressure reduction.

AT_2_R function is highly state‐dependent, varying with tissue type, disease context, and signaling milieu. While AT_2_R activation typically mediates vasodilation under physiological conditions [25], its pathological overexpression in RH may be linked to pressor effects or signaling dysfunction. We observed elevated AT_2_R expression in the refractory hypertensive state, which was paradoxically exacerbated by conventional therapy. CDC effectively downregulated this aberrant AT_2_R expression, correlating with improved blood pressure control. It is crucial to recognize that receptor expression levels do not always directly predict functional activity. Our findings suggest that in this model, AT_2_R overexpression may represent a maladaptive component of a dysregulated RAAS. By normalizing this overexpression, CDC may help re‐establish systemic homeostasis, contributing to the synergistic effect. The in vitro experiment, in which CDC‐containing serum specifically reduced AT_2_R protein in overexpressing endothelial cells, provides direct mechanistic support.

A notable finding was the dissociation between serum Ang II levels and renal cortical AT_1_R/AT_2_R expression patterns, highlighting the complexity of RAAS regulation in RH. First, circulating Ang II may not accurately reflect intrarenal RAAS activity, which is more critical for long‐term blood pressure control. Local tissue Ang II generation, degradation, and receptor binding create a relatively independent microenvironment. Second, our regimen included an AT_1_R blocker (irbesartan), which typically induces a compensatory rise in plasma renin and Ang II levels. This feedback may obscure the treatment’s actual suppressive effect on the RAAS axis. Thus, in this pharmacological context, serum Ang II levels reflect a drug‐altered feedback state rather than directly indicating the underlying pathology or therapeutic efficacy. This underscores the necessity to distinguish systemic from tissue‐specific RAAS activity. Future studies measuring local renal Ang II, renin, or ACE activity will better delineate the precise sites of CDC’s intervention within the RAAS pathway.

### 4.3. Other Potential Pathways: Improvement of Insulin Resistance May Contribute to the Synergistic Effect

Insulin resistance is a significant comorbid mechanism in hypertension [26]. Beyond modulating the RAAS, we found that the CDC combination regimen, especially IAT + CDC, significantly lowered the HOMA‐IR index, indicating improved INS sensitivity. This aligns with the established link between ameliorated INS resistance and enhanced blood pressure control [27]. Thus, improving INS sensitivity may represent an additional pathway contributing to CDC’s synergistic antihypertensive effect, possibly related to the known gluco‐metabolic benefits of its principal herb, *Dendrobium officinale*. Collectively, these findings exemplify the multitarget, multipathway mode of action characteristic of TCM formulations.

### 4.4. Safety and Clinical Translation Prospects

Regarding safety, the constituent herbs of CDC (*Dendrobium officinale* and *Paeonia lactiflora*) are pharmacopoeia‐listed and have a long history of clinical use. Preliminary toxicological studies by our group revealed no significant adverse effects at the tested doses. However, the safety profile, human tolerability, and potential pharmacokinetic interactions of this fixed compound, especially when combined with specific antihypertensive drugs, require systematic evaluation in standardized Phase I/II clinical trials. Future translational studies should also systematically assess potential herb–drug interactions, particularly the pharmacokinetic profiles of CDC when coadministered with standard antihypertensive agents. The strategy of adjunctive CDC therapy proposed here presents a promising integrative medicine approach for managing RH, particularly for enhancing blood pressure stability. Its definitive clinical value, however, awaits confirmation through further research.

## 5. Limitations and Future Directions

This study has several limitations. First, while sample sizes (*n* = 8 for efficacy and *n* = 4 for molecular analysis, with tissues taken from a subgroup to assess post‐treatment persistence) were adequate to demonstrate primary effects, they may lack the power to detect subtle differences in more variable secondary endpoints (e.g., serum Ang II). Second, the focus on molecular and functional outcomes, without histopathological assessment of renal injury (e.g., fibrosis, glomerular morphology), limits insights into target organ protection. Third, the downstream signaling pathways mediating CDC’s effect on AT_2_R and the specific bioactive constituents involved remain unknown. Fourth, we monitored body weight as a general health indicator (Supplementary Table 1). Normotensive Wistar rats gained weight as expected, whereas SHRs with RH maintained a stable, lower weight, highlighting their distinct metabolic state. Although not designed to dissect metabolic determinants, this study did not analyze correlations between weight changes and blood pressure or molecular responses, which is a limitation. Future work should investigate how the metabolic profile of refractory SHRs interacts with combination therapies. Finally, as a preclinical study, translation to humans requires validation in clinical trials.

Future studies should (1) employ sample sizes determined by a priori power analysis based on the effect sizes observed here; (2) utilize radiotelemetry for 24‐h continuous blood pressure monitoring in conscious rats to accurately assess CDC’s impact on blood pressure stability and variability; (3) include histopathological evaluations to determine renoprotective effects; (4) isolate and test individual CDC components to identify active constituents and elucidate their molecular mechanisms, including downstream signaling (e.g., ERK and NO pathways); and (5) initiate systematic clinical trials to evaluate the safety, efficacy, and optimal dosing of CDC as an adjunctive therapy for patients with RH.

## 6. Conclusion

In conclusion, CDC acts synergistically with conventional antihypertensive drugs in a RH rat model, enhancing both the magnitude and stability of blood pressure reduction. This adjunctive therapy significantly improved blood pressure target achievement rates. Mechanistically, the benefits were associated with the attenuation of pathological renal AT_2_R overexpression, revealing a novel pathway for augmenting antihypertensive efficacy. This integrated approach warrants further investigation as a promising strategy for managing clinical RH.

## Author Contributions

Xiaoyu Chen, Jie Wang, Zedong Gong, Yue Wu, Xiaomin Xue, and Tuo Feng: writing–review and editing and methodology. Yingzhi Chen and Shiyong Chen: writing–original draft and investigation. Xiaoming Jin: writing–review and editing and supervision. Shiyong Chen: writing–original draft and formal analysis. Cheng Tong: writing–original draft and software. Zedong Gong: writing–original draft and resources. Xuan Chen and Zeming Ren: validation and resource. Guanhai Dai: project administration and data curation. Yeling Tong: data curation and resource. Xiyu Mei: funding acquisition and supervision. Renzhao Wu: funding acquisition and conceptualization. Based on the contribution assessment, Xiaoyu Chen, Cheng Tong, Jie Wang, Yue Wu, and Tuo Feng should be presented as co‐authors.

## Funding

This study was supported by the Research Institute and Research Project Science and Technology Program of Zhejiang Province (Grant Nos.: 2023TD001 and KJTYSZX2025), the “Sharpshooter and Leading Goose + *X*” Science and Technology Program of Zhejiang Province (Grant No.: 2025C02181), the Zhejiang Provincial Bureau of Traditional Chinese Medicine (Grant No.: 2021ZB077), the Zhejiang Provincial Traditional Chinese Medicine Science and Technology Program (Grant No.: 2026ZL0227), the Zhejiang Basic Public Welfare Research Program (Grant No.: LGF21H270002), and the Institute Specific Foundation of Zhejiang Provincial Department of Science and Technology (Grant No.: YSZX2201).

## Conflicts of Interest

The authors declare no conflicts of interest.

## Supporting Information

This file, “Supporting Tables,” contains 11 tables presenting experimental data on the effects of Compound Dendrobium Candidum combined with antihypertensive agents in refractory hypertensive rats. Details are as follows:

Supporting Table 1. Effects of combined use of three Western antihypertensive agents containing hydrochlorothiazide on the body weight of rats during the refractory hypertensive (RH) screening period (*x* ± *s*, unit: g). Supporting Table 2. Primer sequences of each gene. Supporting Table 3. Effects of combined use of three Western antihypertensive agents containing hydrochlorothiazide on the SBP of rats during the refractory hypertensive (RH) screening period (*x* ± *s*, unit: mmHg). Supporting Table 4. Effect of the combination of CDC and Western antihypertensive agents on SBP in refractory hypertensive (RH) spontaneous hypertensive rats (SHRs) after 6 weeks of treatment (*x* ± *s*, unit: mmHg, *n* = 8; drug withdrawal for 3 days, *n* = 4). Supporting Table 5. Effect of CDC combined with Western antihypertensive agents on serum Ang II content in in refractory hypertensive (RH) spontaneous hypertensive rats (SHRs) at 6 weeks of treatment (*x* ± *s*, unit: pg/mL, *n* = 4). Supporting Table 6. Effect of CDC combined with Western antihypertensive agents on the relative mRNA expression of AT_1_R and AT_2_R in renal cortex of refractory hypertensive (RH) spontaneous hypertensive rats (SHRs) at 6 weeks of treatment (*x* ± *s*, *n* = 4). Supporting Table 7. Effect of CDC combined with Western antihypertensive agents on the relative protein expression of AT_1_R and AT_2_R in renal cortex of refractory hypertensive (RH) spontaneous hypertensive rats (SHRs) at 6 weeks of treatment (*x* ± *s*, *n* = 4). Supplementary Table 8. Effects of CDC combined with Western antihypertensive agents on the serum INS concentration, fasting blood glucose (FGB), and insulin resistance (IR) index of refractory hypertensive (RH) spontaneous hypertensive rats (SHRs) at 6 weeks of treatment (*x* ± *s*, *n* = 4). Supporting Table 9. Effect of CDC combined with Western antihypertensive agents on the relative mRNA expression of ISR, ISR‐α, and ISR‐β in the renal cortex of refractory hypertensive (RH) spontaneous hypertensive rats (SHRs) at 6 weeks of treatment (*x* ± *s*, *n* = 4). Supporting Table 10. Effect of serum and lentivirus on the relative mRNA expression of AT1R and AT2R on day 5 of treatment (*x* ± *s*, *n* = 4). Supporting Table 11. Relative proteins expression of AT1R and AT2R in glomerular endothelial cells on day 5 of treatment with serum and lentivirus (*x* ± *s*, *n* = 3). Supporting Figure S1. Mass Spectrometry Analysis of Compound Dendrobium Candidum (CDC). This file contains the complete UPLC‐Q‐TOF/MS dataset for the chemical characterization of the CDC chewable tablets (batch 201905002), including the total ion chromatogram and fragmentation spectra used to identify the major bioactive constituents.

## Supporting information


**Supporting Information** Additional supporting information can be found online in the Supporting Information section.

## Data Availability

The data that support the findings of this study are available from the corresponding author, Xiaomin Xue, upon reasonable request.
