# Supplementary material for: Synergistic Effects of Compound Dendrobium Candidum and Antihypertensive Medications on Refractory Hypertension in Spontaneously Hypertensive Rats
Source: Cardiol Res Pract. 2026 Mar 11;2026:5582480. doi: 10.1155/crp/5582480 (PMC12977149; doi:10.1155/crp/5582480)

Hydroxytyrosol (Mass/FragMass/RT/Isotope/Library/Formula/Ion Ratio)

Retention Time: 14.96 minutes
Precursor m/z : 155.0703
Fit (%) N/A **RFit (%)** N/A

Exp RT: 14.95 minutes
Analyte Name:
Hydroxytyrosol

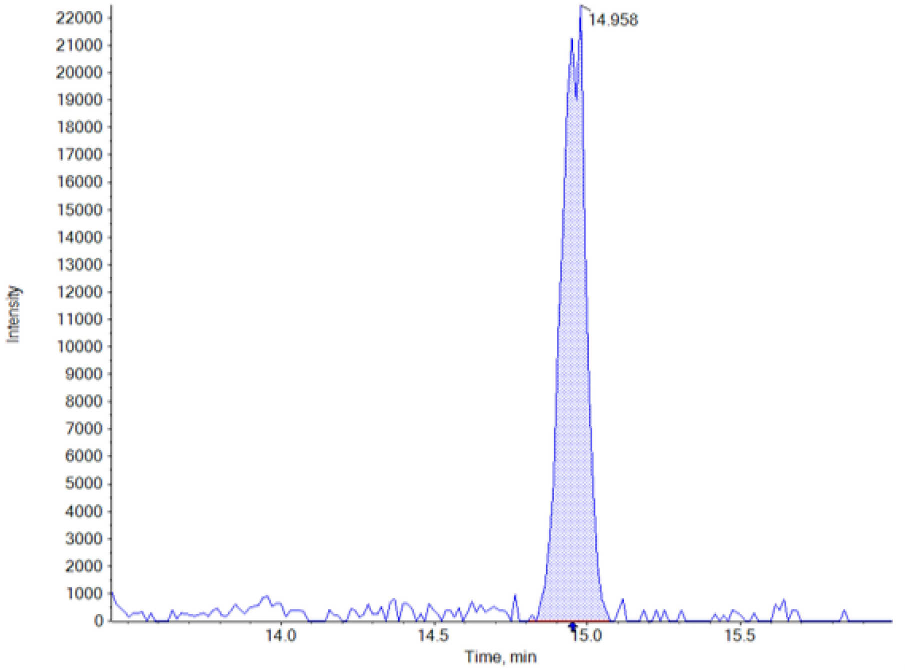

Collision Energy = 35 ± 15 eV

Acquired / Library MSMS

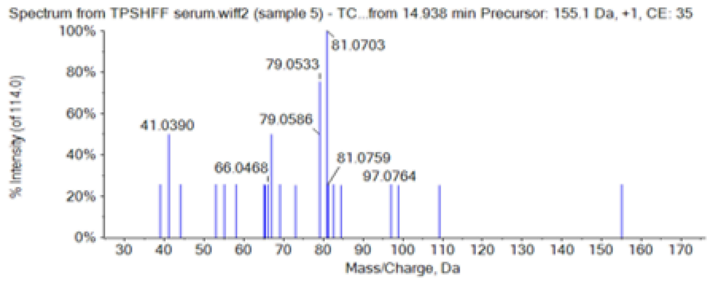

Acquired / Theoretical MS

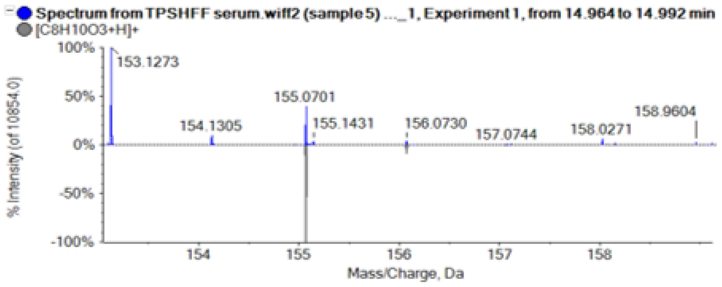

Corilagin (Mass/FragMass/RT/Isotope/Library/Formula/Ion Ratio)

Retention Time: 5.73 minutes
Precursor m/z : 633.0733
Fit (%) N/A **RFit (%)** N/A

Exp RT: 5.74 minutes
Analyte Name:
Corilagin

Collision Energy = 35 ± 15 eV

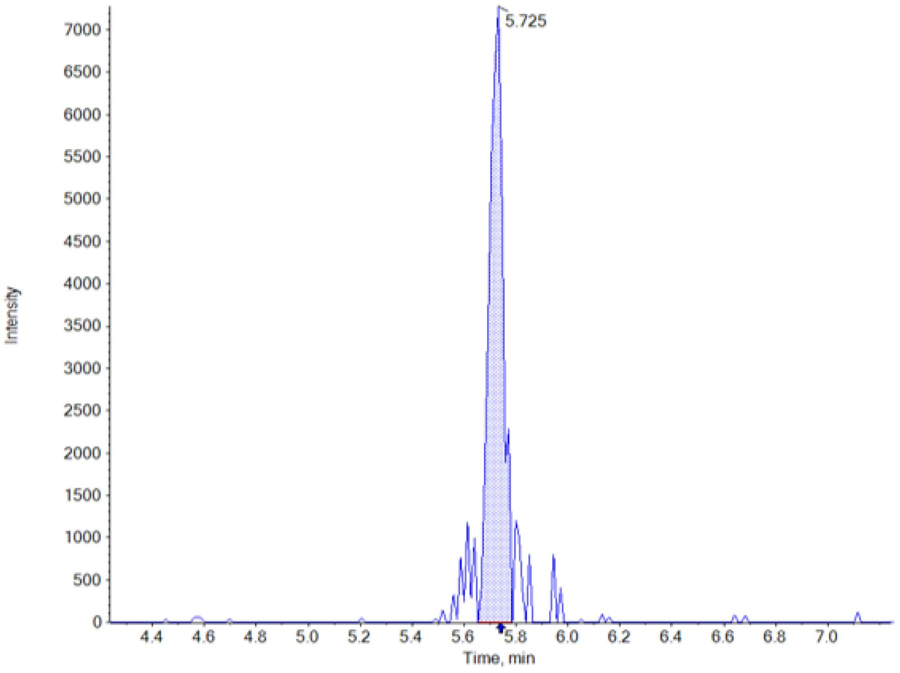

Acquired / Library MSMS

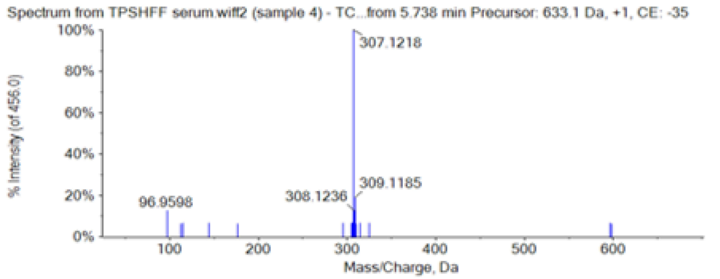

Acquired / Theoretical MS

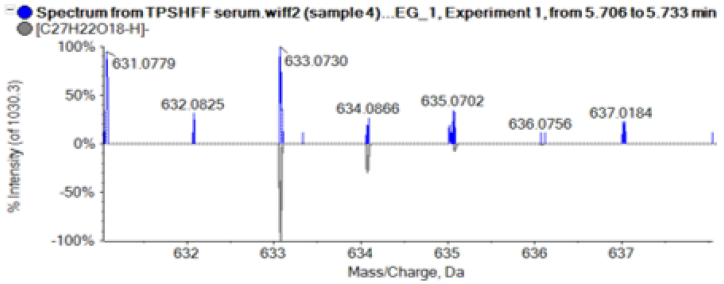

Fumaric acid (Mass/FragMass/RT/Isotope/Library/Formula/Ion Ratio)

Retention Time: 2.00 minutes
Precursor m/z : 115.0037
Fit (%) N/A **RFit (%)** N/A

Exp RT: 2.01 minutes
Analyte Name:
Fumaric acid

Collision Energy = 35 ± 15 eV

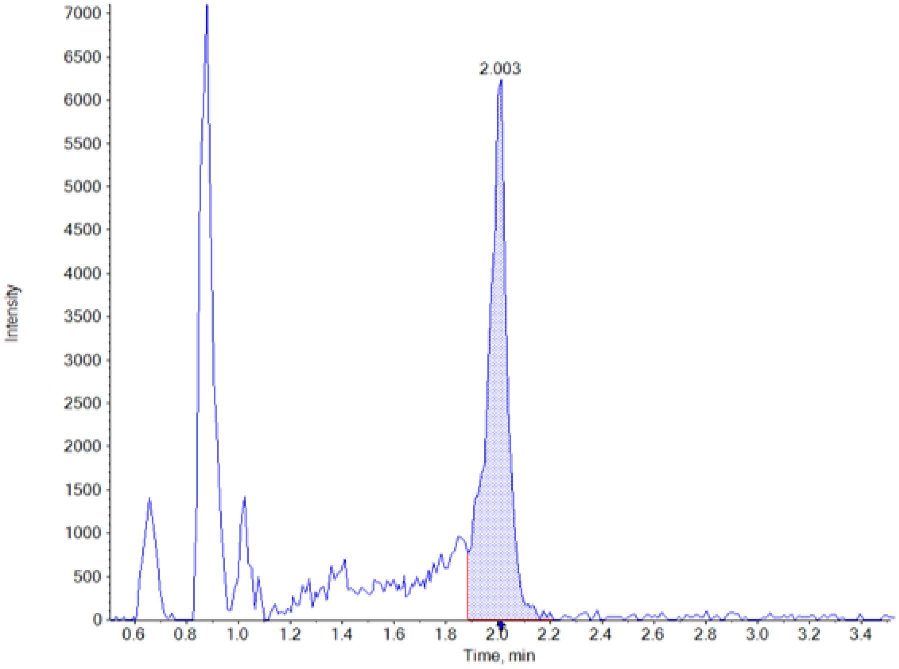

Acquired / Library MSMS

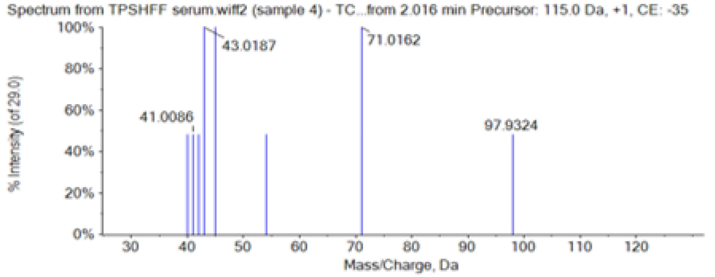

Acquired / Theoretical MS

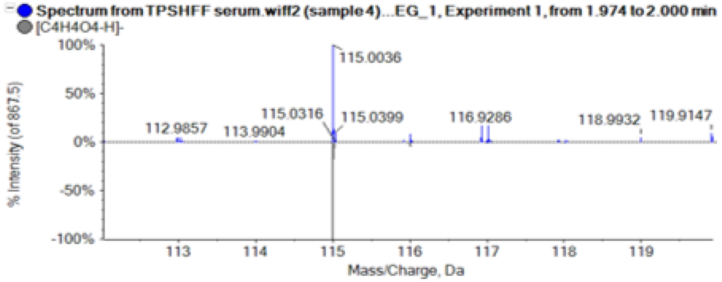

Kaempferide (Mass/FragMass/RT/Isotope/Library/Formula/Ion Ratio)

<table>
<tr><td colspan="2">Retention Time: 12.31 minutes
Precursor m/z : 299.0561
Fit (%) N/A RFit (%) N/A</td><td colspan="2">Exp RT: 12.24 minutes
Analyte Name:
Kaempferide</td></tr>
<tr><td></td><td colspan="3">Collision Energy = 35 ± 15 eV</td></tr>
<tr><td rowspan="2">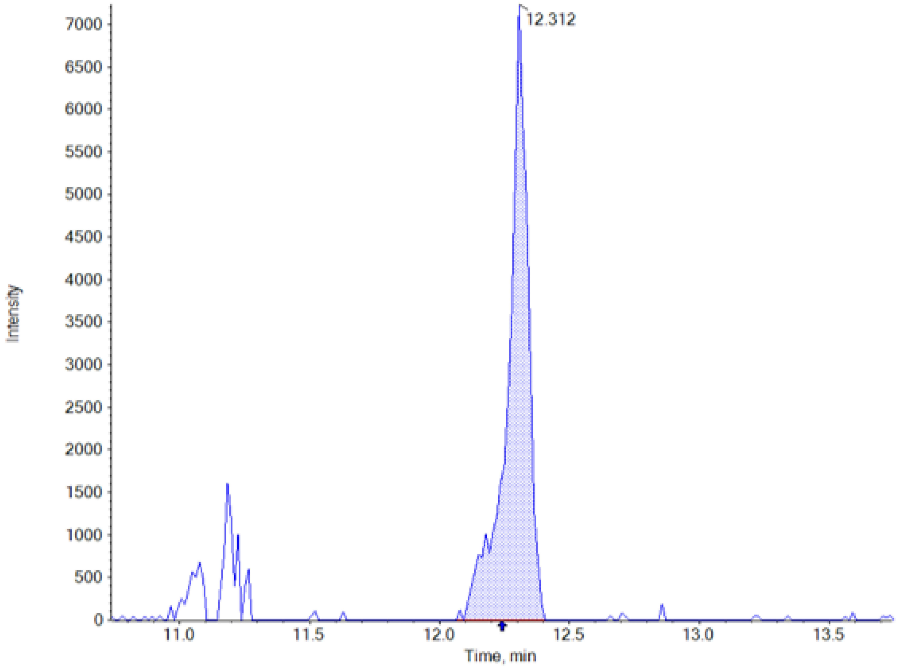</td><td>Acquired / Library MSMS</td><td colspan="2">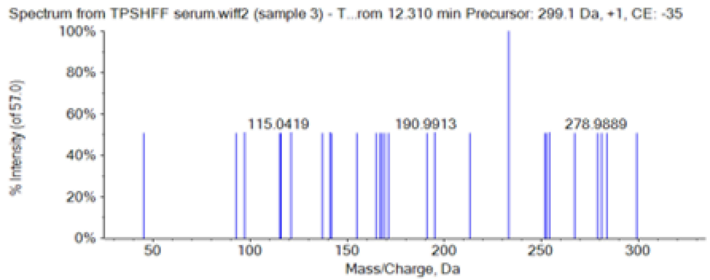</td></tr>
<tr><td>Acquired / Theoretical MS</td><td colspan="2">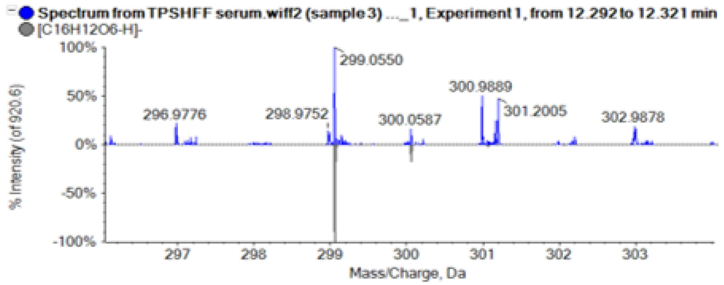</td></tr>
</table>

Isoquercitrin (Mass/FragMass/RT/Isotope/Library/Formula/Ion Ratio)

Retention Time: 11.59 minutes
Precursor m/z : 463.0882
Fit (%) N/A **RFit (%)** N/A

Exp RT: 11.58 minutes
Analyte Name:
Isoquercitrin

Collision Energy = 35 ± 15 eV

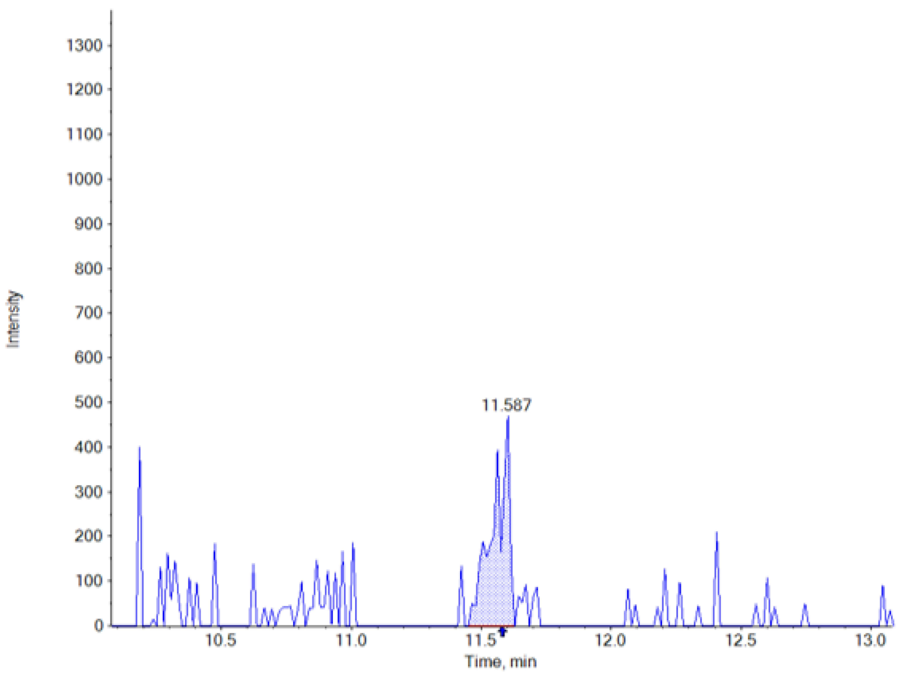

Acquired / Library MSMS

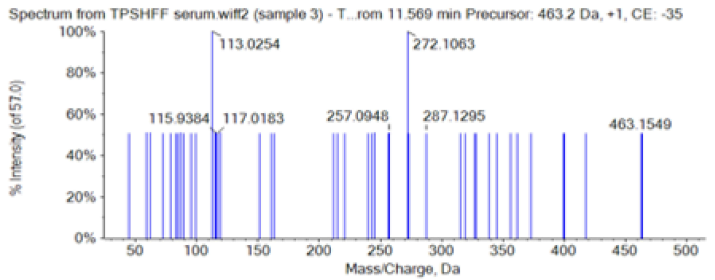

Acquired / Theoretical MS

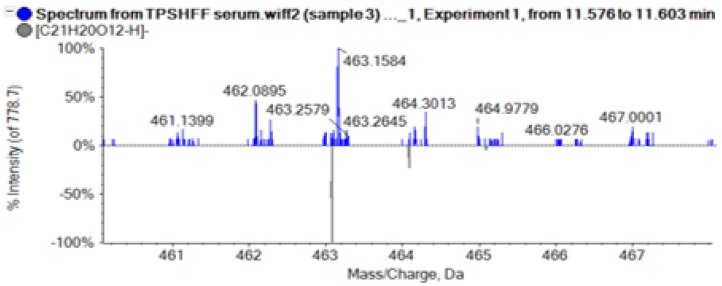

Loureirin A (Mass/FragMass/RT/Isotope/Library/Formula/Ion Ratio)

Retention Time: 8.71 minutes
Precursor m/z : 285.1132
Fit (%) N/A **RFit (%)** N/A

Exp RT: 8.69 minutes
Analyte Name:
Loureirin A

Collision Energy = 35 ± 15 eV

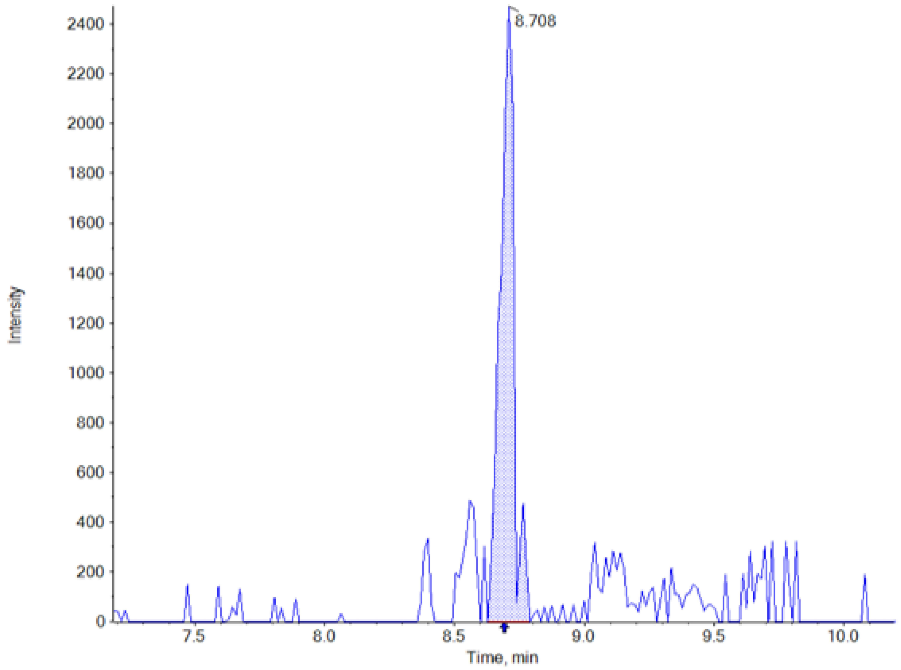

Acquired / Library MSMS

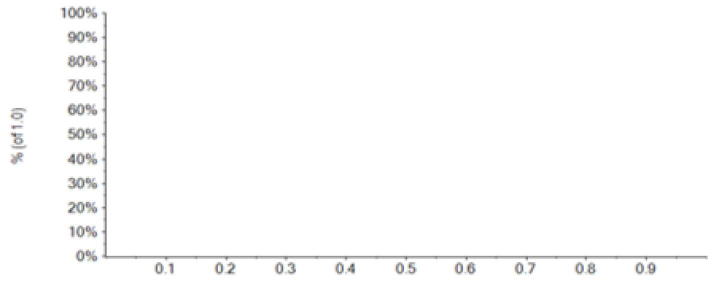

Acquired / Theoretical MS

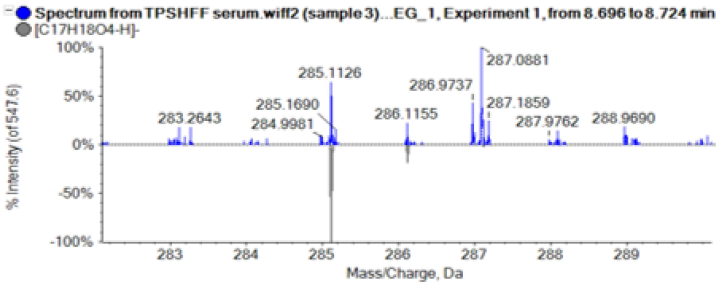

Syringaldehyde (Mass/FragMass/RT/Isotope/Library/Formula/Ion Ratio)

Retention Time: 4.35 minutes
Precursor m/z : 181.0506
Fit (%) N/A **RFit (%)** N/A

Exp RT: 4.34 minutes
Analyte Name:
Syringaldehyde

Collision Energy = 35 ± 15 eV

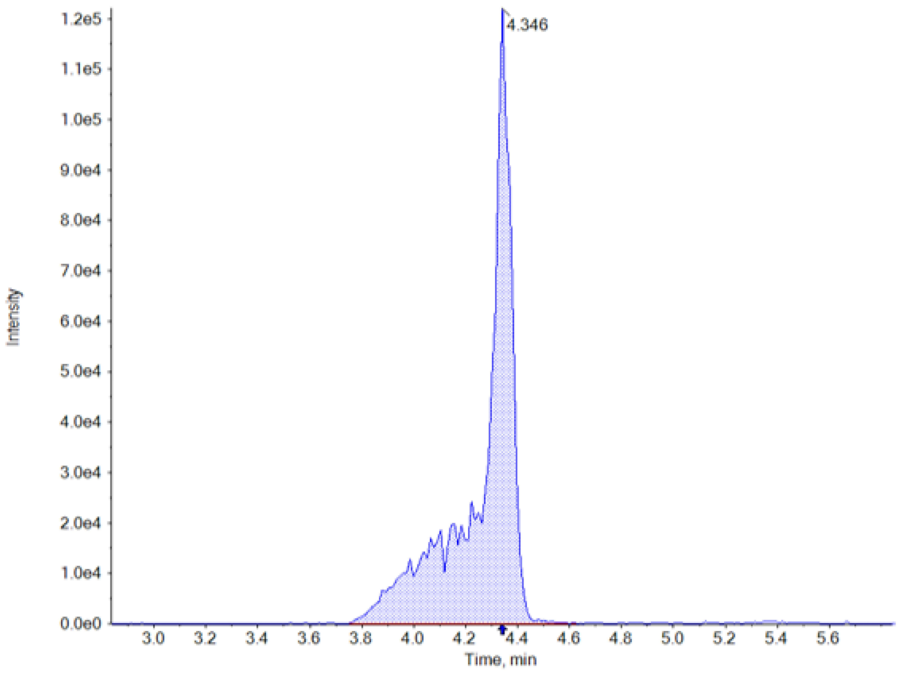

Acquired / Library MSMS

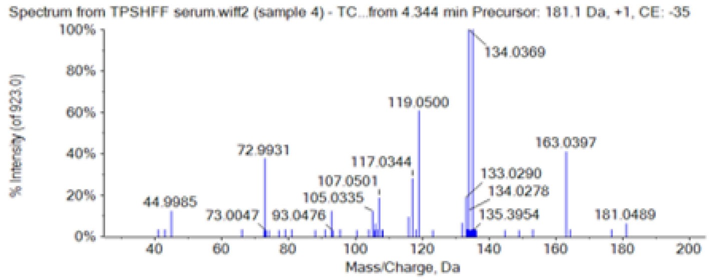

Acquired / Theoretical MS

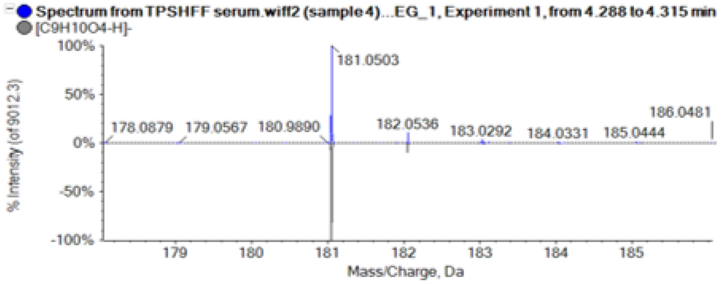

Rutin (Mass/FragMass/RT/Isotope/Library/Formula/Ion Ratio)

Retention Time: 6.55 minutes
Precursor m/z : 609.1461
Fit (%) N/A **RFit (%)** N/A

Exp RT: 6.57 minutes
Analyte Name:
Rutin

Collision Energy = 35 ± 15 eV

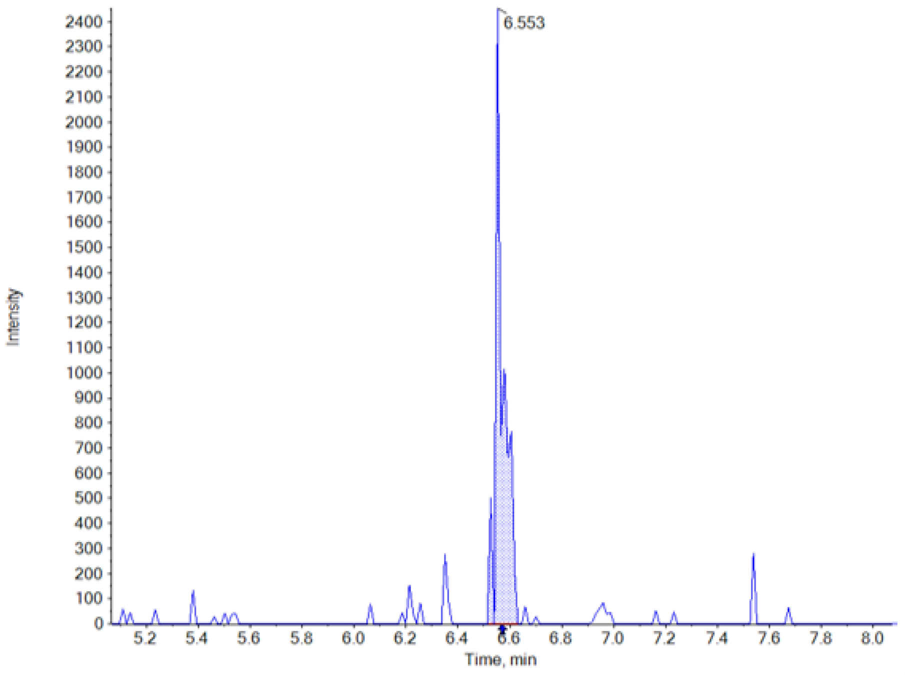

Acquired / Library MSMS

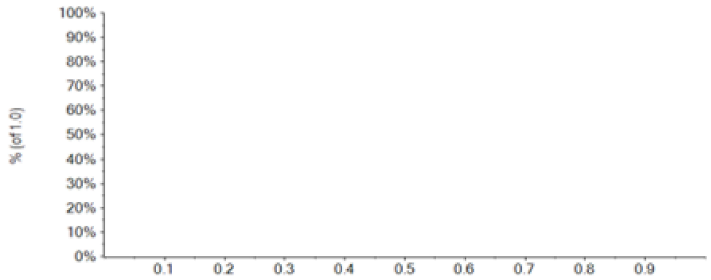

Acquired / Theoretical MS

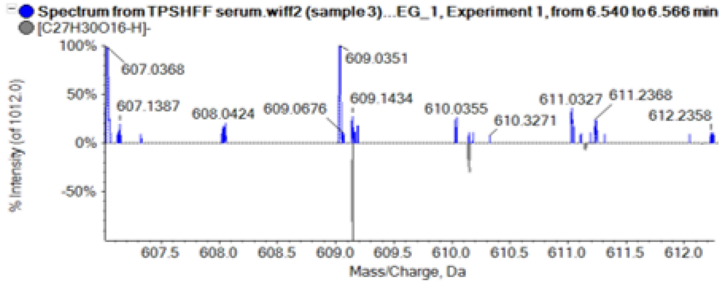

Hyperin (Mass/FragMass/RT/Isotope/Library/Formula/Ion Ratio)

Retention Time: 11.59 minutes
Precursor m/z : 463.0882
Fit (%) N/A **RFit (%)** N/A

Exp RT: 11.58 minutes
Analyte Name:
Hyperin

Collision Energy = 35 ± 15 eV

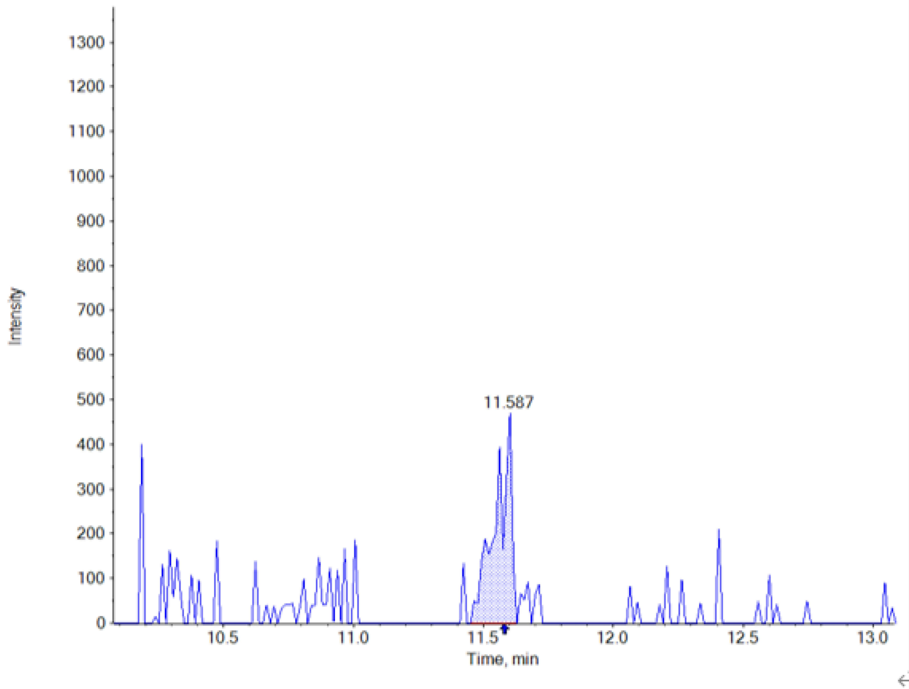

Acquired / Library MSMS

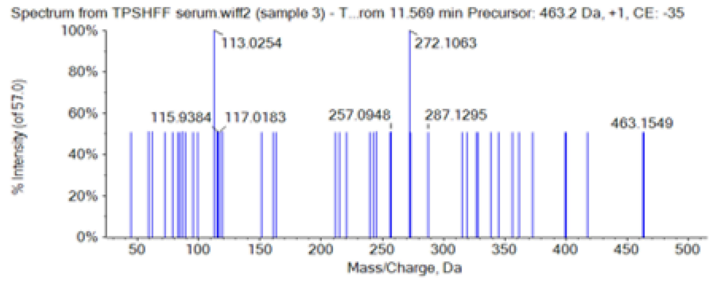

Acquired / Theoretical MS

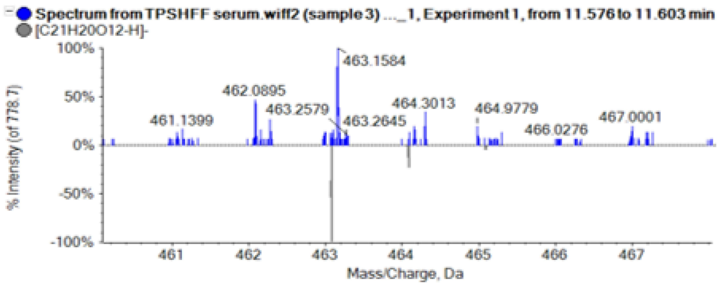

Paeoniflorin (Mass/FragMass/RT/Isotope/Library/Formula/Ion Ratio) ✓ ● ● ✓ ✓ ● ●

Retention Time: 6.31 minutes
Precursor m/z : 479.1559
Fit (%) 95.0% **RFit (%)** 100.0%

Exp RT: 6.32 minutes
Analyte Name:
Paeoniflorin

Collision Energy = 35 ± 15 eV

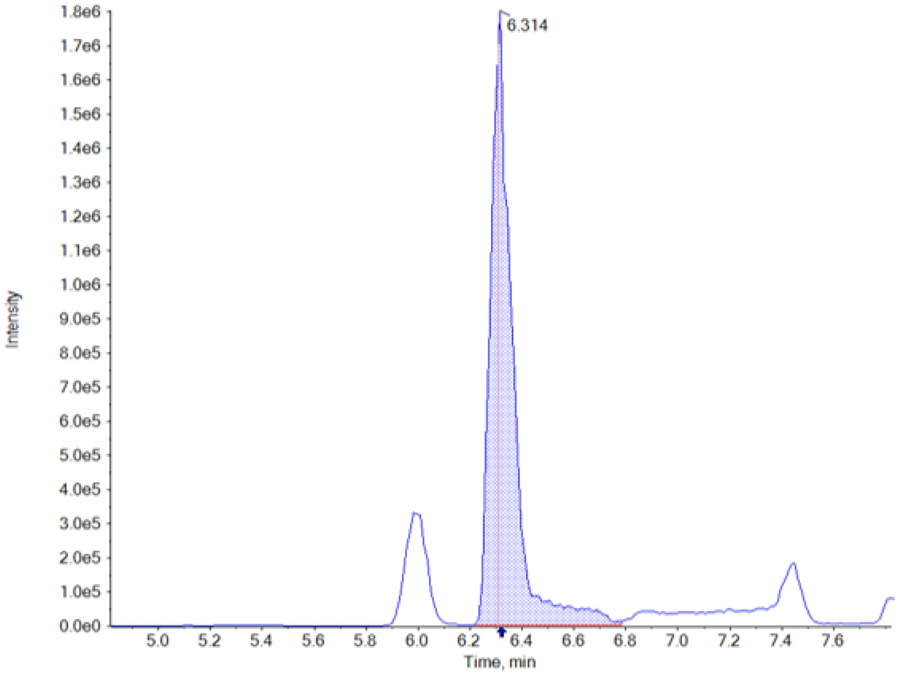

Acquired / Library MSMS

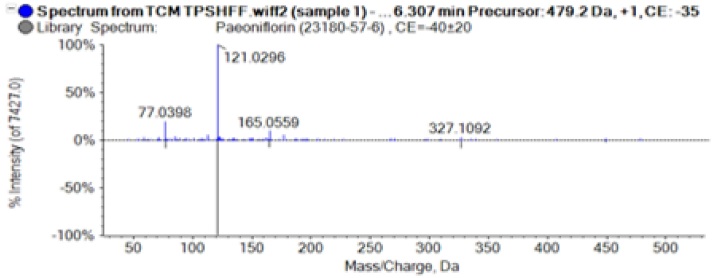

Acquired / Theoretical MS

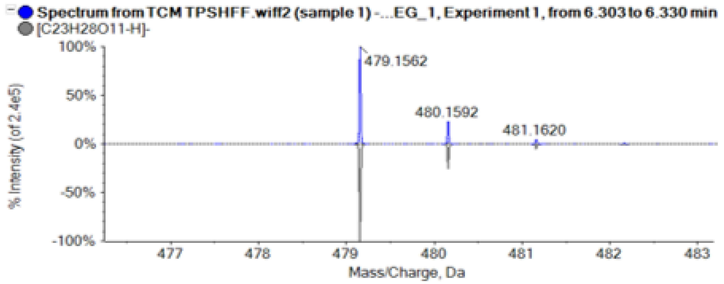

(Mass/FragMass/RT/Isotope/Library/Formula/Ion Ratio)

Retention Time: 11.86 minutes
Precursor m/z : 427.2252
Fit (%) N/A **RFit (%)** N/A

Exp RT: 11.86 minutes
Analyte Name:

Collision Energy = 35 ± 15 eV

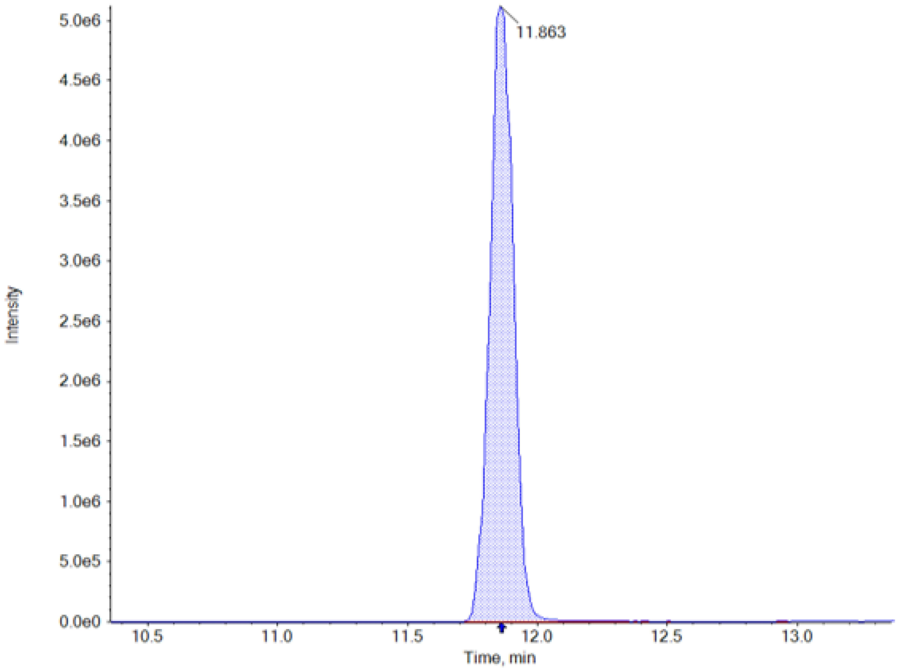

Acquired / Library MSMS

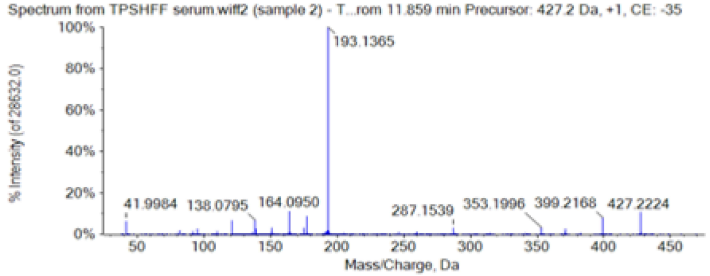

Acquired / Theoretical MS

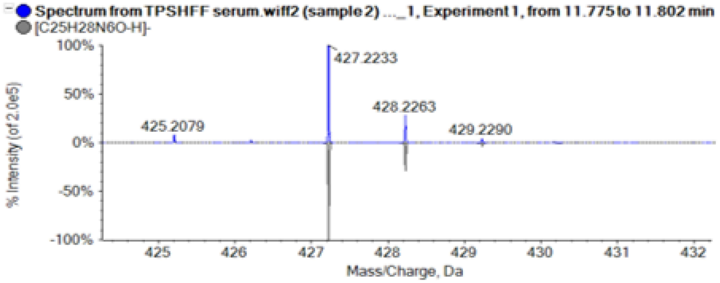

(Mass/FragMass/RT/Isotope/Library/Formula/Ion Ratio)

Retention Time: 4.14 minutes
Precursor m/z : 409.1525
Fit (%) N/A **RFit (%)** N/A

Exp RT: 4.14 minutes
Analyte Name:

Collision Energy = 35 ± 15 eV

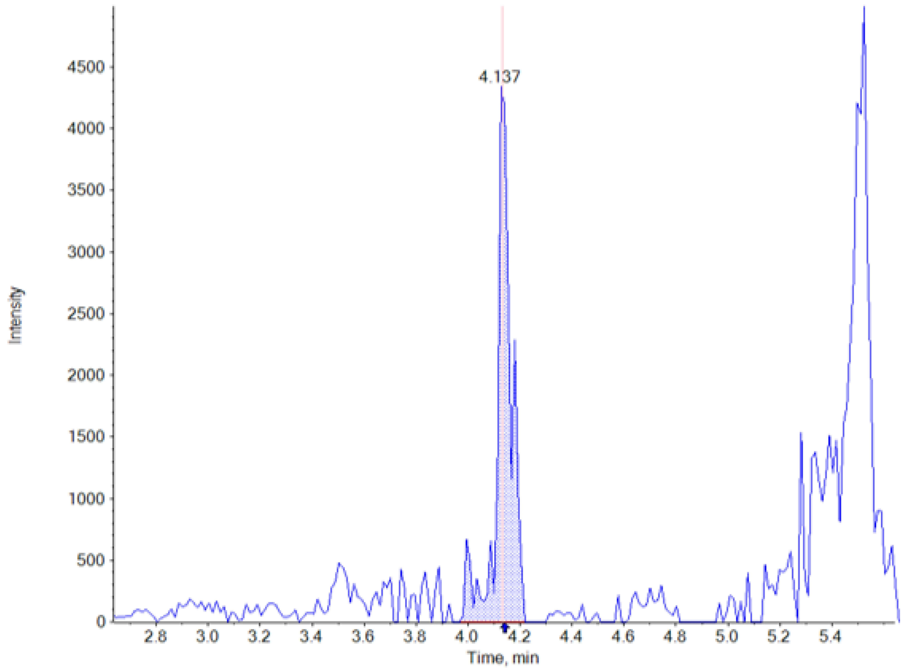

Acquired / Library MSMS

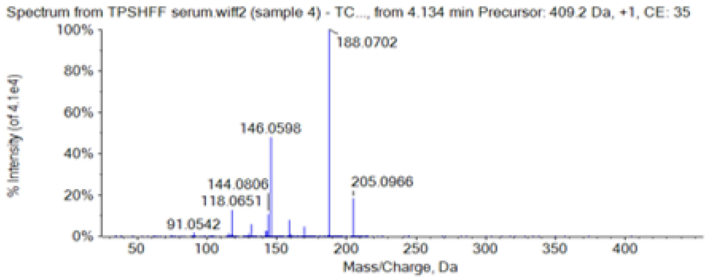

Acquired / Theoretical MS

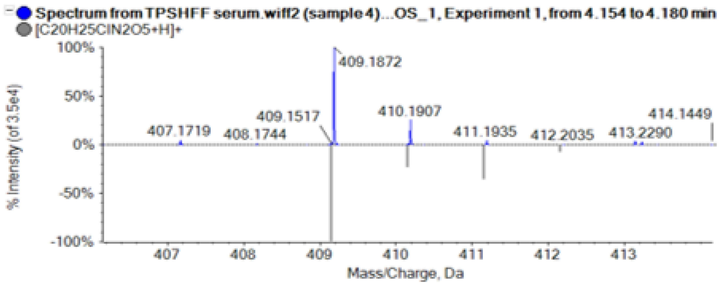

Supplement: Supplementary file 1 — Supporting Information Additional supporting information can be found online in the Supporting Information section. [file CRP-2026-5582480-s001.zip › Supplementary Figure S1.pdf]
