# Supplementary material for: Synergistic Effects of Compound Dendrobium Candidum and Antihypertensive Medications on Refractory Hypertension in Spontaneously Hypertensive Rats
Source: Cardiol Res Pract. 2026 Mar 11;2026:5582480. doi: 10.1155/crp/5582480 (PMC12977149; doi:10.1155/crp/5582480)
Supplement: Supplementary file 1 — Supporting Information Additional supporting information can be found online in the Supporting Information section. [file CRP-2026-5582480-s001.zip › Supplementary Tables.docx]

**Supplementary Tables**

Supplementary Table 1. Effects of combined use of three Western antihypertensive agents containing hydrochlorothiazide on the body weight of rats during the refractory hypertensive (RH) screening period (x ± s, unit: g)

| Groups | Before gavage administration | Administered for 2 weeks | Administered for 4 weeks |
| --- | --- | --- | --- |
| Normal group (n=8) | 570.38±51.83 | 591.50±50.67 | 619.63±58.12 |
| SHR rats group (n=49) | 321.53±24.92^■^ | 321.33±23.06^■^ | 331.96±23.93^■^ |

Note: Compared with the normal group, SHR group: ■*P*<0.01. Each group is 6 months old.

Supplementary Table 2. Primer sequences of each gene

| Gene | Upstream primer sequence | Downstream primer sequence |
| --- | --- | --- |
| AT_1_R | 5’-GCTTCAACCTCTACGCCAGTGTG-3’ | 5’-CAGCCAGATGATGATGCAGGTGAC-3’ |
| AT_2_R | 5’-ACGCAACTGGCACCAATGAGTC-3’ | 5’-TCAGCCACAGCCAGATTGAAGATG-3’ |
| ISR | 5’-GTCGCTCCTATGCTCTGGTGTCA-3’ | 5’-GAGGTTGTGCTTGTTCCAGTCCC-3’ |
| ISR-α | 5’-ATGAACGCCGGACCTATGGA -3’ | 5’-GATCCAGGGGAACGGAAGGA -3’ |
| ISR-β | 5’-GAGAAGATCACCCTCCTCCGAGAG-3 | 5’-CGAAGACTGGCTGACTCATTGACC -3’ |
| β-actin | 5’-GGAGATTACTGCCCTGGCTCCTA-3’ | 5’-GACTCATCGTACTCCTGCCTGCTG-3’ |

Supplementary Table 3. Effects of combined use of three Western antihypertensive agents containing hydrochlorothiazide on the systolic blood pressure of rats during the refractory hypertensive (RH) screening period (x ± s, unit: mmHg).

| Course of treatment | Normal group (n=8) | SHR rats group (n=49) |
| --- | --- | --- |
| Before treatment | 114.30±1.95 | 209.79±3.58^■^ |
| 3 h post-dose after 2 weeks of treatment | 117.50±1.31 | 166.07±3.49^■^ |
| 24 h post-dose after 2 weeks of treatment | 116.86±1.75 | 179.64±4.78^■^ |
| 3 h post-dose after 4 weeks of treatment | 115.01±1.37 | 165.48±3.43^■^ |
| 24 h post-dose after 4 weeks of treatment | 115.55±1.86 | 175.70±3.89^■^ |

Note: Compared with the normal group, SHR group: ■*P*<0.01.

Supplementary Table 4. Effect of the combination of compound dendrobium candidum (CDC) and Western antihypertensive agents on systolic blood pressure (SBP) in refractory hypertensive (RH) spontaneous hypertensive rats (SHRs) after 6 weeks of treatment (x ± s, unit: mmHg, n = 8; drug withdrawal for 3 days, n = 4).

| Course of treatment | Normal group | Model group | CDC group | IA group | IA+CDC group | IAT group | IAT+CDC group |
| --- | --- | --- | --- | --- | --- | --- | --- |
| Screening before treatment | 114.30±1.95^■^ | 210.24±5.56 | 210.28±3.33 | 209.58±3.78 | 209.16±0.65 | 209.61±3.34 | 208.97±3.30 |
| Screening for 4 weeks | 115.55±1.86^■^ | 175.23±2.56 | 175.14±1.68 | 175.85±7.14 | 176.03±1.83 | 175.23±3.08 | 175.19±2.94 |
| 3 h post-dose after 3 weeks of treatment | 114.86±1.32^■^ | 210.23±2.13 | 184.99±2.54^■^ | 184.47±2.13^■^ | 168.25±1.70^■●★◇^ | 161.27±3.61^■●^ | 150.95±5.89^■●◇▼^ |
| 24 h post-dose after 3 weeks of treatment | 114.19±0.79^■^ | 208.76±1.14 | 187.40±2.32^■^ | 190.04±2.62^■^ | 172.74±1.31^■●★◆^ | 182.41±3.22^■^ | 163.84±2.54^■●◆▼^ |
| 3 h post-dose after 6 weeks of treatment | 114.28±0.75^■^ | 211.62±1.00 | 181.79±2.30^■^ | 179.79±2.58^■^ | 166.56±0.81^■●★◆^ | 159.00±2.33^■●^ | 137.23±1.94^■●◆▼^ |
| 24 h post-dose after 6 weeks of treatment | 113.65±0.71^■^ | 211.61±1.58 | 187.22±1.93^■^ | 187.00±3.62^■^ | 171.50±2.19^■●★◆^ | 177.99±2.21^■●^ | 160.42±2.65^■●◆▼^ |
| 3 days after stopping medication | 115.02±2.91^■^ | 209.12±0.60 | 189.76±4.35^■^ | 197.21±7.36^■○^ | 182.83±1.16^■○★◆^ | 191.16±4.34^■^ | 170.18±1.70^■●◆▼^ |

Note: comparison between each group and model group: ■*P*<0.01; comparison between IA group, IAT group, IA+CDC group, IAT+CDC group, and CDC group, respectively: ●*P*<0.01, ○*P*<0.05; comparison between IA+CDC group and IA group: ★*P*<0.01, ☆*P*<0.05; comparison between IA+CDC group, IAT +CDC group, and IAT group, respectively: ◆*P*<0.01, ◇*P*<0.05; comparison between IAT+CDC group and IA+CDC group: ▼*P*<0.01.

Supplementary Table 5. Effect of compound dendrobium candidum (CDC) combined with Western antihypertensive agents on serum angiotensin II (AngⅡ) content in in refractory hypertensive (RH) spontaneous hypertensive rats (SHRs) at 6 weeks of treatment (x ± s, unit: pg/mL, n = 4).

|  | Normal group | Model group | CDC group | IA group | IA+CDC group | IAT group | IAT+CDC group |
| --- | --- | --- | --- | --- | --- | --- | --- |
| Ang Ⅱ | 162.25±100.74 | 242.00±156.24 | 284.58±271.97 | 119.17±57.88 | 683.17±497.52 | 277.42±183.36 | 71.25±45.94^□^ |

Note: compared with the model group: □*P*<0.05.

Supplementary Table 6. Effect of compound dendrobium candidum (CDC) combined with Western antihypertensive agents on the relative mRNA expression of AT_1_R and AT_2_R in renal cortex of refractory hypertensive (RH) spontaneous hypertensive rats (SHRs) at 6 weeks of treatment (x ± s, n = 4).

| Group | AT_1_R | AT_2_R |
| --- | --- | --- |
| Normal group | 0.017357±0.004652^□^ | 0.001622±0.001840 |
| Model group | 0.633032±0.968639 | 0.253351±0.331333 |
| CDC group | 0.677674±0.663013 | 0.216328±0.189156 |
| IA group | 2.111289±1.508037 | 0.873483±0.661673 |
| IA+CDC group | 0.029948±0.004696^○^ | 0.003541±0.003088^○^ |
| IAT group | 1.381978±1.655795 | 0.437260±0.480458 |
| IAT+CDC group | 0.039768±0.031900^○^ | 0.012865±0.021599^○◇^ |

Note: compared with the model group: □*P*<0.05; IA+CDC group, IAT+CDC group compared with CDC group, respectively: ○*P*<0.05; comparison between IAT+CDC group and IAT group: ◇*P*<0.05.

Supplementary Table 7. Effect of compound dendrobium candidum (CDC) combined with Western antihypertensive agents on the relative protein expression of AT1R and AT2R in renal cortex of refractory hypertensive (RH) spontaneous hypertensive rats (SHRs) at 6 weeks of treatment (x ± s, n = 4).

| Group | AT_1_R | AT_2_R |
| --- | --- | --- |
| Normal group | 0.473±0.082 | 0.332±0.104 |
| Model group | 0.491±0.033 | 0.441±0.069 |
| CDC group | 0.535±0.062 | 0.344±0.153 |
| IA group | 0.596±0.128 | 0.465±0.147 |
| IA+CDC group | 0.321±0.104^□*^ | 0.262±0.042^□*^ |
| IAT group | 0.554±0.084 | 0.451±0.138 |
| IAT+CDC group | 0.522±0.071 | 0.414±0.097 |

Note: comparison between IA+CDC group and model group: □*P*<0.05; comparison between IA+CDC group and IA group: **P*<0.05.

Supplementary Table 8. Effects of compound dendrobium candidum (CDC) combined with Western antihypertensive agents on the serum insulin concentration, fasting blood glucose (FGB), and insulin resistance (IR) index of refractory hypertensive (RH) spontaneous hypertensive rats (SHRs) at 6 weeks of treatment (x ± s, n = 4).

| Group | Insulin concentration  (unit: uIU/mL) | FGB  (unit: mmol/L) | IR index |
| --- | --- | --- | --- |
| Normal group | 38.41±26.10 | 5.25±0.21 | 8.80±5.64 |
| Model group | 38.41±25.78 | 4.90±0.70 | 8.22±5.82 |
| CDC group | 18.58±12.56 | 5.25±0.34 | 4.28±2.79 |
| IA group | 21.67±13.80 | 5.50±0.52 | 5.47±3.81 |
| IA+CDC group | 13.49±6.10 | 5.13±0.21 | 3.04±1.31 |
| IAT group | 16.88±7.89 | 5.00±0.47 | 3.65±1.54 |
| IAT+CDC group | 11.08±2.68 | 4.90±0.41 | 2.39±0.52^□^ |

# Note: compared with the model group: □*P*<0.05.

Supplementary Table 9. Effect of compound dendrobium candidum (CDC) combined with Western antihypertensive agents on the relative mRNA expression of ISR, ISR-α, and ISR-β in the renal cortex of refractory hypertensive (RH) spontaneous hypertensive rats (SHRs) at 6 weeks of treatment (x ± s, n = 4).

| Group | ISR | ISR-α | ISR-β |
| --- | --- | --- | --- |
| Normal group | 0.012222±0.002877 | 0.001508±0.000563 | 0.002194±0.001070 |
| Model group | 0.010969±0.007265 | 0.000911±0.000845 | 0.137583±0.214442 |
| CDC group | 0.016642±0.008498 | 0.000441±0.000228 | 0.262927±0.267647 |
| IA group | 0.005065±0.006858 | 0.000487±0.000899 | 0.815370±0.596322 |
| IA+CDC group | 0.014711±0.001339 | 0.002623±0.001699^○^ | 0.004799±0.002262^○^ |
| IAT group | 0.008235±0.008039 | 0.000635±0.000572 | 0.456758±0.526483 |
| IAT+CDC group | 0.016684±0.003545 | 0.001325±0.000699^○^ | 0.009835±0.013040^○^ |

Note: IA+CDC group, IAT+CDC group compared with CDC group, respectively: ○*P*<0.05.

Supplementary Table 10. Effect of serum and lentivirus on the relative mRNA expression of AT_1_R and AT_2_R on day 5 of treatment (x ± s, n = 4).

| Group | AT_1_R | AT_2_R |
| --- | --- | --- |
| Normal group | 0.000961±0.000209^□^ | 0.000013±0.000006^□^ |
| Model group | 0.002968±0.000907 | 0.000089±0.000042 |
| 0.5% drug-free serum group | 0.013773±0.020674 | 0.000198±0.000165 |
| 0.5% medicated serum group | 0.013565±0.020038 | 0.000181±0.000219 |
| AT_2_R KN group | 0.030602±0.018001 | 0.000543±0.000395 |
| AT_2_R K group | 0.009299±0.014282 | 0.000028±0.000019^○^ |
| AT_2_R K+0.5% medicated serum group | 0.013154±0.018609 | 0.000152±0.000155 |
| AT_2_R ON group | 0.022395±0.018407 | 0.000288±0.000263 |
| AT_2_R O group | 0.015603±0.022634 | 0.050677±0.042966^☆^ |
| AT_2_R O+0.5% medicated serum group | 0.010802±0.014058 | 0.030676±0.036046 |

Note: comparison between the model group and the normal group: □*P*<0.05; comparison between the AT_2_R K group and the AT_2_R KN group: ○*P*<0.05; comparison between the AT_2_R O group and the AT_2_R ON group: ☆*P*<0.05.

Supplementary Table 11. Relative proteins expression of AT_1_R and AT_2_R in glomerular endothelial cells on day 5 of treatment with serum and lentivirus (x ± s, n = 3).

| Group | AT_1_R | AT_2_R |
| --- | --- | --- |
| Normal group | 0.304±0.020^■^ | 0.269±0.022^■^ |
| Model group | 0.436±0.031 | 0.579±0.012 |
| 0.5% drug-free serum group | 0.315±0.046 | 0.166±0.072^*^ |
| 0.5% medicated serum group | 0.321±0.086 | 0.193±0.057^◆^ |
| AT_2_R KN group | 0.429±0.106 | 0.235±0.049 |
| AT_2_R K group | 0.186±0.008^○^ | 0.137±0.014^○^ |
| AT_2_R K+0.5% medicated serum group | 0.181±0.102 | 0.112±0.040 |
| AT_2_R ON group | 0.153±0.026 | 0.116±0.013 |
| AT_2_R O group | 0.491±0.161 | 0.411±0.038^◆^ |
| AT_2_R O+0.5% medicated serum group | 0.174±0.028 | 0.139±0.022^▲^ |

Note: comparison between the model group and the normal group: ■*P*<0.01; comparison between the 0.5% drug-free serum group and model group: **P*<0.01; comparison between the 0.5% medicated serum group and model group: ◆*P*<0.01; comparison between the AT_2_R K+0.5% medicated serum group, AT_2_R K group, and AT_2_R KN group: ○*P*<0.05; comparison between the AT_2_R O group and AT_2_R ON group: ◆*P*<0.01; comparison between the AT_2_R O+0.5% medicated serum group and AT_2_R O group: ▲*P*<0.01.
